# Breaking down the silos of artificial intelligence in surgery: glossary of terms

**DOI:** 10.1007/s00464-022-09371-y

**Published:** 2022-06-21

**Authors:** Andrea Moglia, Konstantinos Georgiou, Luca Morelli, Konstantinos Toutouzas, Richard M. Satava, Alfred Cuschieri

**Affiliations:** 1 Department of Translational Research and New Technologies in Medicine and Surgery, University of Pisa, Pisa, Italy; 2 1st Propaedeutic Surgical Unit, Hippocrateion Athens General Hospital, Athens Medical School, National and Kapodistrian University of Athens, Athens, Greece; 3 Department of General Surgery, University of Pisa, Pisa, Italy; 4 Department of Surgery, University of Washington Medical Center, Seattle, WA USA; 5 Scuola Superiore Sant’Anna of Pisa, 56214 Pisa, Italy; 6 Institute for Medical Science and Technology, University of Dundee, Dundee, DD2 1FD UK

**Keywords:** Artificial intelligence surgery, Machine learning surgery, Deep learning surgery

## Abstract

**Background:**

The literature on artificial intelligence (AI) in surgery has advanced rapidly during the past few years. However, the published studies on AI are mostly reported by computer scientists using their own jargon which is unfamiliar to surgeons.

**Methods:**

A literature search was conducted in using PubMed following the preferred reporting items for systematic reviews and meta-analyses (PRISMA) statement. The primary outcome of this review is to provide a glossary with definitions of the commonly used AI terms in surgery to improve their understanding by surgeons.

**Results:**

One hundred ninety-five studies were included in this review, and 38 AI terms related to surgery were retrieved. Convolutional neural networks were the most frequently culled term by the search, accounting for 74 studies on AI in surgery, followed by classification task (*n* = 62), artificial neural networks (*n* = 53), and regression (*n* = 49). Then, the most frequent expressions were supervised learning (reported in 24 articles), support vector machine (SVM) in 21, and logistic regression in 16. The rest of the 38 terms was seldom mentioned.

**Conclusions:**

The proposed glossary can be used by several stakeholders. First and foremost, by residents and attending consultant surgeons, both having to understand the fundamentals of AI when reading such articles. Secondly, junior researchers at the start of their career in Surgical Data Science and thirdly experts working in the regulatory sections of companies involved in the AI Business Software as a Medical Device (SaMD) preparing documents for submission to the Food and Drug Administration (FDA) or other agencies for approval.

**Supplementary Information:**

The online version contains supplementary material available at 10.1007/s00464-022-09371-y.

Artificial intelligence (AI) is a branch of computer science enabling computers to build mathematical models to train and solve problems by mimicking human cognition, provided these models have been trained properly [1]. Machine learning (ML) is a subfield of AI that enables computers to make predictions based on underlying data patterns, while deep learning (DL) is a subset of ML [2] (Fig. 1).

**Fig. 1:**
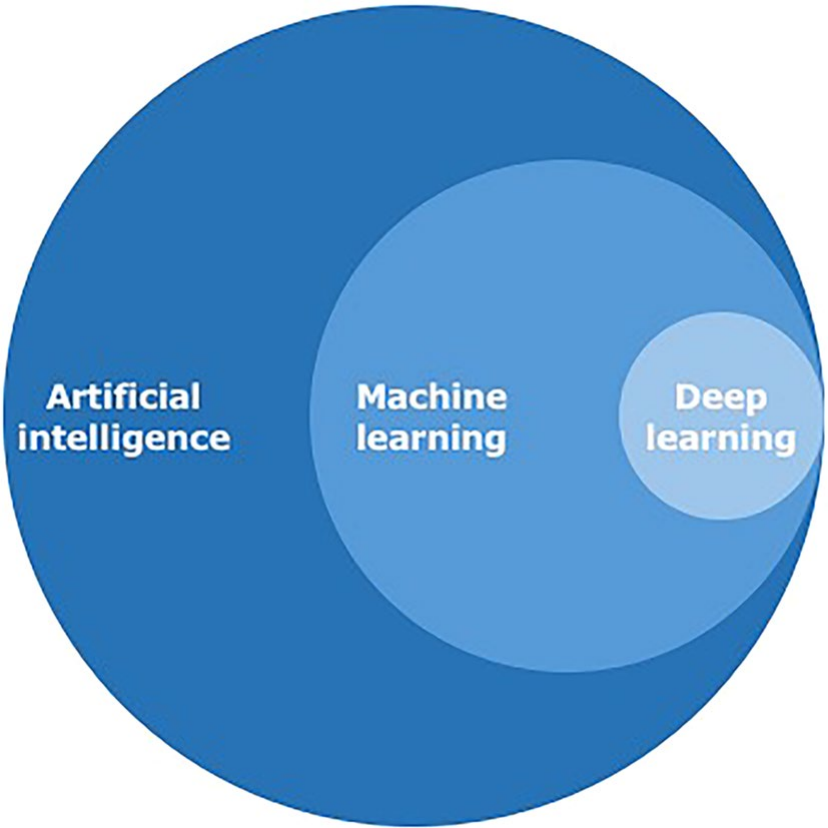
Relationship among artificial intelligence, machine learning and deep learning

AI has been applied successfully to medicine during the last years [3]. Surgical Data Science is the research field of AI, specifically targeted towards surgical practice to improve the quality of surgical healthcare delivery through the capture, organization, analysis, and modeling of data [4]. AI can be applied to surgery (i) to obtain an automated objective assessment of skills during training [5, 6], (ii) to automate surgery following the paradigm of self-driving cars with respect to robot-assisted surgery (RAS) [7], (iii) to enable intra-operative navigation [8] and (iv) to detect early and thereby prevent errors and improve patient outcomes [9].

As literature on AI in surgery increases, surgeons should be able to understand the published studies on AI. Although this additional AI knowledge may be perceived currently as too theoretical and not useful, and consequently overlooked as not essential for good practice, it is more than likely that in the future this attitude will change as medical devices based on AI will enter the market and be integrated into the clinical practice.

However, this is not a trivial task as the AI terms used in these studies are generated by computer scientists using their own jargon which is largely unfamiliar to surgeons. Thus, the widespread clinical use of AI in surgery faces several challenges. These include AI algorithms that are not transparent or “understood” by surgeons who for this reason regard AI systems as ‘black box’ in nature and understood incompletely [10]. Indeed, few physicians have the necessary knowledge to understand them [11]. Additionally, the data files structure are often extremely complex [12].

Thus, the primary outcome of this review is to provide definitions of the commonly used AI terms in surgery to simplify their understanding by surgeons. In this way, we want to contribute to the development of a multidisciplinary collaboration between surgeons, engineers, and computer scientists. The secondary outcome is to provide, in a supplement, a detailed list of surgical articles in which AI terminology is used.

## Materials and methods

A literature search was conducted in September 2021 on PubMed following the preferred reporting items for systematic reviews and meta-analyses (PRISMA) statement [13]. This search required several steps. First, we retrieved any AI terms used in laparoscopy or RAS. We then compiled an initial database using the following search strategy:

1. ((((artificial intelligence) OR (machine learning)) OR (deep learning)) OR (computer vision)) OR (Natural language processing)
2. ((((laparoscopy) OR (robotic)) OR (minimal invasive)) OR (minimally invasive)) OR (laparoscopic)
3. (surgery) OR (surgical)
4. #2 AND #3
5. #1 AND #4
6. #5 NOT REVIEW

**Fig. 2:**
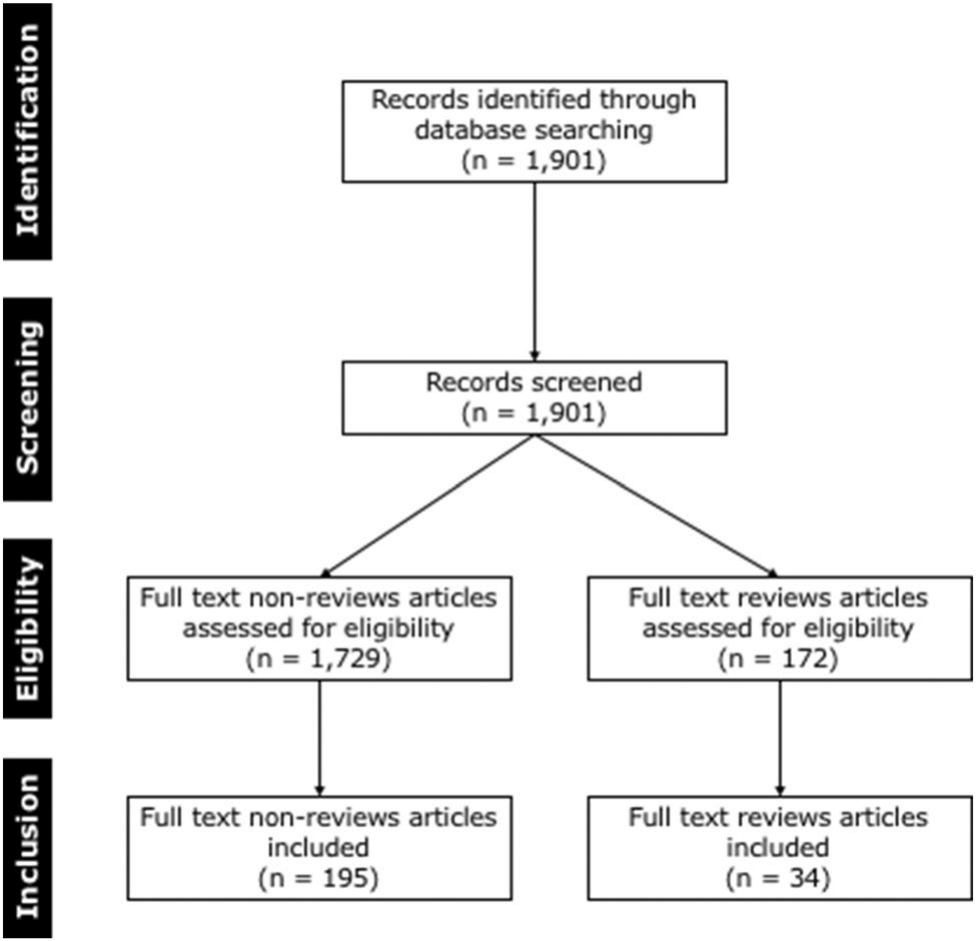
Flow diagram of the search and inclusion process

We then applied the following filters to the retrieved articles: Abstract, Journal Article, in the last 5 years, Humans, and English language. Thus, in total 1729 articles were retrieved and two reviewers (AM and KG) independently screened titles and abstracts of all identified publications for relevance before inclusion. The exclusion criteria were reviews, letters, non-peer reviewed articles, conference abstracts, and proceedings. A total of 195 articles were finally included in our initial database. Data from these articles were extracted and checked by two authors (AM and KG). During the second step, we compiled a list of AI terms mentioned at least once in the abstracts of our initial database. The three fundamental terms i.e., AI, ML, and DL were excluded. A final database based on the resulting 38 AI-related terms was established. Next, we then conducted a reciprocal number of queries to our initial database to establish the frequency each term was used. In the third step, we searched for the first mention of each AI term in the literature. In this step, we performed a query in our primary search using item #5 tagged as review to identify all published reviews. We applied the same filters as those used in the initial search for non-review articles and retrieved 34 review articles. The same methodology as in the first step was followed by the same authors (AM and KG) who inspected all reviews to see whether they provided explanations of any of the 38 AI terms either into text or in the online Appendix. Ten published reviews in the last five years contained at least one of the 38 AI terms. The flowchart of the searches performed, based on PRISMA statement is shown in Fig. 2.

## Results

Table 1 depicts the list of the 38 AI terms and their occurrence in our initial search (second column). The final number of occurrences used in our final database is shown in the third column. Table 1 shows that convolutional neural networks (CNNs) were the most frequent, appearing in 74 studies, followed by classification in 62, artificial neural networks (ANNs) in 53, and regression in 49. The most frequent expressions were supervised learning (reported in 24 articles), support vector machine (SVM) in 21, and logistic regression in 16. The rest of the 38 terms were mentioned infrequently. The detailed list of the citations occurring in each one of the 38 terms is shown in the online Appendix.

**Table 1:** Occurrence of 38 main AI terms used in Surgery

	Term	Number of occurrences	Number of final occurrences
1	AdaBoost	3	3
2	Artificial neural networks (ANNs)	104	53
3	Autoencoder	4	4
4	Classification	105	62
5	Clustering	22	12
6	Convolutional neural networks (CNNs)	93	74
7	Decision trees	5	5
8	Dimensionality reduction	3	3
9	Dynamic time warping (DTW)	1	1
10	Ensemble learning	15	11
11	Feed forward neural network	2	2
12	Fully convolutional networks	14	11
13	Gated recurrent units (GRUs)	1	1
14	Generative Adversarial Networks (GAN)	11	7
15	Gradient boosting	3	2
16	Hidden Markov models (HMMs)	6	6
17	Imitation learning	3	3
18	Instance segmentation	10	10
19	JHU-ISI Gesture and Skill Assessment Working Set (JIGSAWS) dataset	26	18
20	*k*-nearest neighbors (*k*NN)	6	6
21	Kernel	3	3
22	Lasso regression	1	1
23	Logistic regression	38	16
24	Long short-term memory (LSTM)	18	18
25	Multilayer perceptrons	4	4
26	Object detection	15	14
27	Principal component analysis	5	5
28	Random Forests	21	15
29	Recurrent neural networks (RNNs)	10	8
30	Regression	95	49
31	Reinforcement learning	7	7
32	Representational learning	18	15
33	Ridge regression	1	1
34	Semantic segmentation	11	11
35	Supervised learning	35	24
36	Support vector machines (SVM)	26	21
37	Transition state clustering (TSC)	1	1
38	Unsupervised learning	12	12

Table 2 reports the occurrence of each one of the 38 main AI terms in the ten retrieved reviews. Except the one by Zhou et al. [14] where 21 out of the 38 (55%) terms were mentioned, only a minority of the terms occurred in the rest, ranging from 12 (32%) [15] to just one (3%) [9].

**Table 2:** Main AI terms mentioned in surgery-related reviews

	Term	Garrow et al. [15]	Chang et al. [21]	Chen et al. [22]	Egert et al. [9]	Hashimoto et al. [23]	Ma et al. [24]	Zhou et al. [14]	Tanzi et al. [25]	Anteby et al. [17]	Van Amsterdam [26]
1	AdaBoost			Y							
2	Artificial neural networks (ANNs)	Y		Y	Y	Y	Y				
3	Autoencoder							Y	Y		
4	Classification	Y	Y	Y		Y	Y	Y	Y		
5	Clustering			Y		Y		Y			
6	Convolutional neural networks (CNNs)	Y	Y	Y		Y	Y	Y	Y	Y	Y
7	Decision trees	Y		Y				Y			
8	Dimensionality reduction										
9	Dynamic time warping (DTW)	Y						Y			
10	Ensemble learning										
11	Feed forward neural network										
12	Fully convolutional networks							Y			
13	Gated recurrent units (GRUs)						Y				Y
14	Generative Adversarial Networks (GAN)							Y	Y		
15	Gradient boosting										
16	Hidden Markov models (HMMs)	Y						Y		Y	Y
17	Imitation learning							Y			
18	Instance segmentation										
19	JHU-ISI Gesture and Skill Assessment Working Set (JIGSAWS) dataset						Y		Y		Y
20	*k*-nearest neighbors (*k*NN)			Y			Y				
21	Kernel	Y		Y				Y			
22	Lasso regression										
23	Logistic regression			Y		Y	Y				
24	Long short-term memory (LSTM)	Y					Y	Y	Y		Y
25	Multilayer perceptrons										
26	Object detection		Y						Y		
27	Principal component analysis						Y	Y	Y		
28	Random Forests	Y	Y	Y		Y	Y	Y			Y
29	Recurrent neural networks (RNNs)	Y	Y			Y	Y	Y	Y		Y
30	Regression			Y		Y		Y			
31	Reinforcement learning					Y	Y	Y	Y		
32	Representational learning										Y
33	Ridge regression										
34	Semantic segmentation		Y								
35	Supervised learning	Y				Y		Y	Y		
36	Support vector machines (SVM)	Y	Y	Y			Y	Y		Y	Y
37	Transition state clustering (TSC)							Y			Y
38	Unsupervised learning					Y		Y			Y

Table 3 provides a glossary with the definitions of the AI terms identified in our search results. A detailed list of surgical articles in which each AI term is included is reported in the online Appendix.

**Table 3:** Glossary of AI terms in surgery

Activation function	A non-linear function applied to a linear function representing neurons. The resulting function is non-linear and allows neural networks to approximate complex phenomena. The most used activation functions are: hyperbolic tangent (tanh), sigmoid, and rectified linear unit (ReLU) [27]
AdaBoost	Short for Adaptive Boosting. It is an ensemble method aggregating several weak learners into a strong one. It first trains a model, e.g. a Decision Tree for classification, to make predictions on the training set. It then increases the relative weight of misclassified training instances. Then it trains a second model with the updated weights and makes predictions on the training set. This time the model makes better predictions. The process is repeated for any additional models [27]
Annotation	The process of drawing the contours of the objects inside an image
Area Under the Curve	It is the area under the receiver operating characteristic (ROC) curve. It returns a value ranging from zero to one [28]. It is used for binary classification
Artificial neural networks (ANNs)	Inspired by biological neurons, these algorithms consist of several layers, each of which includes neurons. Neurons between layers are connected by parameters called weights
Autoencoder	A neural network based on an encoder and a decoder
Backpropagation	An algorithm used to train ANNs. Batches of instances traverse the layers of the ANN until an output is computed (forward pass). The difference between the predicted output and real output is computed. It is the error and called loss. Then, the algorithm computes how much each intermediate layer contributed to the error. The algorithm then measures how much of the error contributions came from each connected neuron in the previous layers, working until the algorithm reaches the input layer (backward pass). During the backward pass, the gradients of the error through all the connections in the ANN are computed. The algorithm then adjusts the weights of the ANN through a gradient descent. The process of forward and backward passes continues until the end of training [29, 30]
Batch normalization	A technique to solve the issue of vanishing/exploding gradient. This operation zero-centers and normalizes each input, then it scales and shifts the result [29, 31]
Bounding box	A box enclosing an object in an image. It is used for computer vision tasks, like detection and segmentation
Classification	A typical supervised learning task to predict a target class (a discrete value) [29]
Clustering	This task aims to identify similar instances and assigning them to clusters, or groups of similar instances. It belongs to unsupervised learning
Convolutional neural networks (CNNs)	A type of neural networks made up of convolutional layers. They are typically used for analysis of images. In convolutional neural networks a small matrix (called filter or kernel) slides over a larger matrix (e.g. an image which can be described as a 2D matrix or tensor of pixels). The convolution is performed by multiplying the filter pixelwise by the portion of the image and summing the result. [32, 33]
Cost function	A function evaluating the model. It computes the difference between the predicted and actual value. Some of the most popular cost functions are: mean absolute error and the mean squared error (for regression), and binary cross entropy and categorical cross entropy (for classification)
Cross-validation	A method to evaluate generalization of models. The most common type of cross-validation is k-fold cross-validation where data are split into parts of equal size, called folds. A model is first trained using the first fold as test set and the other four as training set. Accuracy is evaluated on the first fold. Then, a second model is built using the second fold as test set and the others as training set. Accuracy is evaluated on the second fold. This process is repeated for all k folds. At the end we get an accuracy value for each fold [28]
Decision trees	These ML models use a hierarchy of if/else question leading to a decision. The purpose is to reach the right answer by asking the minimum number of if/else questions [28]. Decision trees look for the best test for each node. They are used for both classification and regression tasks. They tend to overfit the data
Decoder	A neural network decompressing a representational vector back to the original domain [34]
Deep learning (DL)	A subfield of machine learning based on artificial neural networks [32]
Dimensionality reduction	A machine learning technique to reduce significantly the number of features. It is especially useful when the number of features is so high that some problems seem initially unsolvable [29]**.** Dimensionality reduction enables to solve efficiently those problems. Then, ML algorithms can be applied after dimensionality reduction without the risk to run out of computer resources. One of the most popular examples is Principal Component Analysis [35]
Dropout	A common regularization technique. At every training step each neuron has a probability of being ignored, but it may become active again during the next step [30, 34]
Dynamic time warping (DTW)	A technique to dynamically compare time series of data when the time indices between comparison data points do not sync up perfectly. The time series are ‘‘warped” or matched, together based on their similarities at each time point [15]
Encoder	It is a network compressing high-dimensional input data into a lower-dimensional representational vector [32]
Ensemble	The process of learning from an aggregation of models [29]. For instance, random forests are an ensemble of decision trees
Exploding gradient	A phenomenon in which the gradient of the cost function with respect to each parameter becomes so big causing the weights to receive large updates. This way the training diverges [29]
False negative (FN)	Ratio of positive instances which are incorrectly classified as negative [29]
False positive (FP)	Ratio of negative instances which are incorrectly classified as positive [29]
Features	Independent variables acting as input to the model. In images a feature corresponds to the value of the color channel (e.g. RGB) of each pixel
Federated learning	A method to train an AI model across multiple decentralized devices on servers holding local data, without exchanging them
Feed forward neural network	A type of ANNs where the signal flows only in one direction, from the input to the output [29]
Floating-point operations per second (FLOPS)	A measure of computers performance used in computations with floating-point numbers
Fully connected layers	In fully connected layers each neuron of one layer is connected to all neurons of the next layers, as in multilayer perceptrons
Fully convolutional networks	A neural network consisting only of convolutional layers [29] in contrast with conventional CNNs which include both convolutional and fully connected layers
Gate Recurrent Unit (GRU)	Gate Recurrent Unit (GRU) is a simplified version of LSTM where the forget and input gates are replaced by reset and update gates. In a GRU there is no output gate [36, 37]
Generative Adversarial Imitation Learning (GAIL)	A GAN based learning method to imitate experts’ behavior. The discriminator learns to distinguish generated performances from expert demonstrations, whereas the generator attempts to mimic the expert to fool the discriminator into thinking as its performance was an expert demonstration [38]
Generative Adversarial Network (GAN)	A type of ANNs with competing networks, called generators and discriminators. The generator takes a random distribution and outputs some data, e.g. an image. The discriminator takes as input either a fake image from the generator or a real image from the training set and must guess if it is real or fake [29, 39]
Gradient boosting	An ensemble model. Like AdaBoost it corrects its predecessors. Gradient Boosting tries to fit the new model to the residual errors made by the previous one [29]
Gradient descent	A popular algorithm to tune the parameters to minimize a cost function. Gradient descent measures the gradient of the cost function with regard to a parameter vector. It goes in the direction of descending gradient. Once the gradient is zero, the minimum of the cost function is reached [29]
Graphics processing unit (GPU)	A chip for parallel computation which results in performance boost for task requiring intensive workload. For this reason, they are used to accelerate AI tasks. A GPU is faster than a central processing unit (CPU)
Grid search	A method to adjust the hyperparameters of supervised models for the best generalization performance [28]
Hidden Markov models (HMMs)	A statistical tool that models a system as a Markov process, which is a system existing in a series of distinct states, with transitions between them occurring at random intervals. In a HMM the states of the model are not directly observable [40]
Hyperparameters	They are parameters which are not estimated from the data. They are used to tune the model parameters
Imitation learning	Also called “learning from demonstration”, it enables robots to perform autonomously new tasks [14]
Instance segmentation	A task of computer vision to predict object instances using segmentation mask
JHU-ISI Gesture and Skill Assessment Working Set (JIGSAWS)	A publicly available RAS dataset collected through a collaboration between the Johns Hopkins University (JHU) and Intuitive Surgical, Inc. (ISI) [41]
K-means clustering	A clustering algorithm splitting a set of samples into k groups by minimizing the variation within the cluster [42]
K-nearest neighbors (k-NN)	A simple ML algorithm considering the k closest points to the point of interest. For classification tasks, the occurrence of the class of each neighbor is counted and the most frequent class is then assigned to the prediction [28]. For a regression task, the prediction is the average value of the neighbors [28]
Kernel	In ML a kernel is a function capable to perform the product between two vectors. There are different types of kernels: linear, polynomial, Gaussian RBF, and sigmoid [29]. In CNNs a kernel is a small matrix sliding over a larger one (e.g. an image). It is also named filter
Lasso regression	A type of regression to regularize linear regression. It is also called L1 regularization. It forces some weights of the features to be zero, which means that some features are ignored by the model [28]
Latent space	A low dimensional space which is mapped to a high-dimensional space. It is used for representational learning
Layer	Neurons in ANNs are grouped in layers. The first layer is called input layer, the last is called output layer. Neurons of one layer are connected to the neuron of the preceding layer and subsequent layer. There are different types of layers: dense or fully connected, convolutional, deconvolutional, pooling, and recurrent
Linear regression	A linear model making predictions by computing the weighted sum of the input features plus a bias term (called also intercept) [29]
Logistic regression	An algorithm used for binary classification. It computes the probability that an instance belongs to a class [29]. If the estimated probability is greater than 50%, then the model predicts that this instance belongs to that class (called positive). Otherwise, it predicts that it belongs to the negative class [29]
Long Short-Term Memory (LSTM)	A type of RNN specialized in remembering information for a long period of time and not suffering from the vanishing gradient and short memory issues of RNNs [43]
Machine learning (ML)	ML, a subfield of AI, is the field of study that gives computers the ability to learn without being explicitly programmed [29]
Model parameters	They are parameters which can be estimated from the data
Multilayer Perceptron (MLP)	A MLP consists of layers of fully connected neurons. The first layer is called input layer, the last output layer, while the internal ones hidden layers [29]
Natural language processing (NLP)	A computer science field focused on helping computers to understand human language
Object detection	A computer vision task consisting of localization and classification
Optimizer	An algorithm used to tune the value of the parameters (i.e., the weights) of an ANN to minimize the cost function
Overfitting	A common behavior of ML models performing well on the training data, but not well on unseen data, i.e., the test data [29]
Padding	A trick used in CNN to have a layer with the same width and height of the previous layer. It requires to add zeros around the inputs [29]
Perceptron	One of the simplest ANNs where each input has a weight [29]
Precision	The accuracy of positive predictions [29]
Precision recall curve	A curve plotting precision and recall for different probability threshold. It is used for binary classification
Principal component analysis (PCA)	A technique for dimensionality reduction. PCA starts by first finding the axis (direction) which accounts for the largest amount of variance of the data. The second axis is orthogonal to the first and accounts for the largest amount of remaining variance, and so on [29]
Random Forests	A type of ensemble ML models. It consists of decision trees that can be used for both classification and regression
Recall	The ratio of the positive instances that are correctly detected by the classifier [29]. It is also called true positive rate or sensitivity [29]
Receiver operating characteristic (ROC) curve	A curve displaying true positive rate (recall) versus false positive rate [29]. It is used for binary classification
Recurrent Neural Network (RNN)	A network made similar to a feedforward network but with connections pointing backward. RNNs are made up of layers of recurrent neurons which receive an input, compute an output and send the output back to them. RNNs have two limitations: vanishing gradient and a limited memory. Both these drawbacks can be solved by LSTMs [29]
Region Proposal Network (RPN)	A fully CNN taking an image as input and outputting bounding boxes and objectness score (i.e., whether an object is present in an image or not). RPN is an essential component of Faster R-CNN for object detection [44]
Region of interest (RoI)	An area of an image which may contain an object
Regression	A task to predict a continuous numeric value [29]
Regularization	A technique to constrain a model to reduce overfitting [29]
Reinforcement learning	A type of ML where the learning system is called agent which can observe an environment, choose and perform actions, and get rewards or penalties [29, 44]
Representational learning	A type of learning where the samples are modeled in a low dimensional latent space instead of the original high dimensional space [32]
Ridge regression	A type of regression to regularize linear regression. It is also called L2 regularization. It forces the weights of the features to be close to zero, thus minimizing their effect on the result
Semantic segmentation	A computer vision task whose goal is to label each pixel of image with a class. It is different form instance segmentation since it does not distinguish instances of the same class
Sensitivity	Another term for recall [29]
Specificity	It is equal to true negative rate, the ratio of negative instances which are correctly classified as negative [29]
Stride	In CNN the kernel (filter) slides over an image. Stride defines the number of pixels the filter slides over the image horizontally and vertically
Spectral Clustering	A clustering method that uses eigenvectors of a matrix derived from the data [28]
Supervised learning	A type of learning where the training set includes the desired solution, called label [29]
Support Vector Machine (SVM)	A model computing a line called decision boundary to separate classes (for classification). For regression, SVM tries to fit as many instances as possible on a stripe while limiting margins violation
Tensor	A multidimensional array or matrix, commonly used in DL
Tensor Processing Unit (TPU)	A chip specifically designed to process tensors. It is faster than a GPU
Testing set	The part of data to see how the model performs on unseen (new) data [28]
Training set	The part of data used to build the model [28]
Transfer learning	A method in which a pretrained model developed for a task is reused as starting point for another task
Transition state clustering (TSC)	An unsupervised algorithm exploiting repeated demonstrations of a task by clustering segment endpoints across demonstrations [45]. TSC complements any motion-based segmentation algorithm by identifying candidate transitions, clustering them by kinematic similarity, and then correlating the kinematic clusters with available sensory and temporal feature [45]
True negative (TN)	Ratio of negative instances which are correctly classified as negative [29]
True positive (TP)	Ratio of positive instances which are correctly classified as positive [29]
True positive rate	Another term for recall [29]
Underfitting	A model which does not perform well in both training data and test data. It occurs typically when the model is too simple. Possible solutions to underfitting include the selection of a more complex algorithm, the use of better features, or the reduction of regularization [29]
Unsupervised learning	A type of learning where the training set is unlabeled, and the system tries to learn without a teacher. An example of unsupervised learning is clustering
Validation set	The part of data to select the parameters of the model [28]
Vanishing gradient	During training of ANNs the gradient of the cost function with respect to each parameter becomes too small so that the weights do not change. This way training does not converge [29]
Visual odometry	A technique to localize a robot by using only a stream of images acquired from a single or multiple cameras attached to the robot [46]

## Discussion

Surgery is in its fourth generation (open surgery, endoluminal surgery, laparoscopic surgery, and RAS). Laparoscopic surgery and RAS provide huge amount of data which can be processed by AI, e.g., one minute of a high-resolution minimal access operations generate 25 times the amount of data found in a high-resolution computed tomography scan [16]. However, minimal access surgery poses a significant challenge to image analysis due to changes in illumination, unfocused frames, blood and smoke in the surgical field, and anatomical diversity [17]. In addition to data from videos, RAS generates data from robot kinematics and event data (e.g., pressing camera and/or clutching pedals) [18].

Since the number of AI terms is increasing constantly with constant expansion of the reported literature on AI, the new generation of surgeons will be required to become familiar with AI knowledge and its reported literature, since AI is expected to have a significant impact on surgery at all the stages: pre-operative, intra-operative, and post-operative.

In this report, we aimed to review and attempt to categorize the relevant terms as well as provide a glossary for surgeons. Our search revealed that CNNs is the AI term reporting the highest number (*n* = 74) of published studies in surgery. This is not surprising as CNNs constitute the backbone of AI frameworks for different applications of computer vision, namely classification (prediction of the correct class of objects in an image), and object detection (localization of objects in addition to classifying the correct class). CNNs also form the basis of complex DL architectures like U-Net for semantic segmentation (definition of pixel-wise borders of objects of the same class) and Mask R-CNN for instance segmentation (definition of pixel-wise borders of each object). As shown in Table 1, all these frameworks have been applied to surgery. When coupled with recurrent neural networks (RNNs), CNNs are capable not only to process spatial information to localize surgical tools, but also to analyze temporal information so that they can be used to analyze the surgical workflow, for instance for action recognition (e.g., dissection and cutting) and phase recognition (e.g., incision of splenorenal ligament). The two common tasks in a ML i.e., classification and regression, were ranked highly in terms of their occurrence. As previously, classification is part of other computer vision tasks e.g., detection and segmentation. Regression is defined as the task involved in the prediction of continuous numerical values. The simplest type of regression is linear regression in which a fitting line is used to model the data, representing the relation between one dependent and one independent variable. There are also more complex types of regression, e.g., non-linear when a curve is used to fit the data or multiple regression, when the dependent variables is related by more than one independent variable. The main ML algorithms were developed for both classification and regression e.g., support vector machine (SVM), random forests, and multilayer perceptrons (MLP) (Table 1).

In contrast there are some AI terms reporting very low number of occurrences, but which are expected to grow rapidly over the next few years. Examples include imitation learning and reinforcement learning, respectively, mentioned in three and seven studies, which may become more common in the near term in wake of the widespread use of robots in several fields, including surgery.

Our glossary provides a comprehensive list of definitions of AI terms to help different stakeholders. Firstly, residents and surgeons with the need to understand the fundamentals of AI while reading articles. Secondly, young researchers starting their career in Surgical Data Science. Thirdly, experts working in the regulatory department of companies in the business of AI Software as a Medical Device (SaMD) to prepare and submit documents for approval from Food and Drug Administration (FDA) or other agencies.

Our glossary contains not only the definitions of AI terms to develop software e.g., the models, but also those related to the hardware necessary to perform the heavy computation requested by AI, e.g., graphical processing unit (GPU). The availability of cloud services hosting large numbers of GPUs significantly lowered the economic barriers to access powerful hardware to train and test even the most complex AI models.

The access to high performance computers at a reasonable price and the possibility to record and store videos of minimal access surgeries would suggest that building and training large datasets is within the reach of most research centers. However, training of AI models for surgery is extremely labor intensive since the process of annotating images (called annotation) requires specific knowledge. It is not a simple annotation of images for “cat versus dog” classification or detection tasks, but rather a process to correctly identify the surgical tools, the anatomical parts (e.g., organs and vessels), and the clinically meaningful events. While laypersons and crowd annotators can reach the same level of surgeons for annotating surgical tools, to identify the anatomy and the quality of a dissection experts surgeons are required [19]. Additionally, the files must be anonymized to protect patients’ identity. Consequently, the size of the datasets of the published studies is typically small. An attempt to overcome this limitation is the Critical View of Safety Challenge [20] of the AI Task Force of the Society of American Gastrointestinal and Endoscopic Surgeons (SAGES), an online platform where it is possible to donate videos of laparoscopic cholecystectomy and contribute as annotators of the videos.

## Conclusions

Surgical data science was recently introduced as the specific field of AI in surgery. Literature on this subject is expanding rapidly. For this reason, there is the need for surgeons to become familiar with the AI terms which were traditionally coined by computer scientists. In this review, we prepared a glossary with definitions of AI terms in surgery after reviewing the literature. This glossary will be useful not only to surgeons, but also to young researchers approaching the field, and companies developing SaMD applications.

## Supplementary Information

Below is the link to the electronic supplementary material.

Supplementary file1 (DOCX 329 kb)
