# Supplementary material for: Breaking down the silos of artificial intelligence in surgery: glossary of terms
Source: Surg Endosc. 2022 Jun 21;36(11):7986–97. doi: 10.1007/s00464-022-09371-y (PMC9613746; doi:10.1007/s00464-022-09371-y)
Supplement: Supplementary file 1 — Supplementary file1 (DOCX 329 kb) [file 464_2022_9371_MOESM1_ESM.docx]

**Appendix**

**Occurrence of AI terms in Surgery related articles**

|  | **Term** | **Articles where the term appears** |
| --- | --- | --- |
| 1 | **AdaBoost** | 1. Prediction of surgeon’s experience [1] 2. Minimally Invasive Optically Guided Surgery [2] 3. Monitoring radiofrequency ablation depth [3] |
| 2 | **Artiﬁcial neural networks (ANNs)** | 1. Video Archiving and Communication System (VACS) [4] 2. AI CC Diagnosis Using Clinical Data [5] 3. Intraoperative safety [6, 7, 7–11] 4. Automated microsurgery analysis [12] 5. MIE cost [13] 6. Visual analysis in LS [14] 7. Vision-Based Suture Tensile Force [15] 8. Computer vision in surgery [16] 9. Predict Changes in Contaminated Bone Cements [17] 10. MIS image synthesis [18] 11. Shoulder muscle activation pattern recognition [19] 12. Invasiveness classification of PA Micropapillary patterns prediction [20] 13. Automated computer vision in POEM [21] 14. Automated LCRS workflow recognition [22] 15. Neurohybrid memristive chip [23] 16. Prediction model for hypotension after induction of anesthesia [24] 17. Retrospective PM detection in GC [25] 18. Prediction of surgical site infection [26] 19. Real-time surgical instrument detection in RAS [27, 28] 20. Spinal Stenosis Grading in MRI [29] 21. Breakthrough Detection Method for Bone Drilling [30] 22. TTTS Inter-fetus membrane segmentation [31] 23. Assessment of bowel preparation [32] 24. Morbidity and mortality prediction after LVSG [33] 25. Force classification during robotic interventions [34] 26. TMJOA diagnosis [35] 27. Operative Steps Recognition in LVSG [9] 28. Image formation for X-ray-based procedures [36] 29. Prediction of early complications of OAGB [37] 30. Urinary continence recovery prediction after RARP [38] 31. Workflow detection and of expert level in laparoscopic suturing and knot-tying [39] 32. Bone milling state identification [40] 33. Invasion depth of the GC based on conventional endoscopy [41] 34. Laparoscopic skills classification using Apple watch motion signals [42] 35. Estimation of grip force and jaw angle on a da Vinci surgical tool using back-end sensors alone [43] 36. Real and predicted mortality under health spending constraints in Italy [44] 37. Neuromuscular interface to operate external robotic devices [45] 38. Intra-operative (shape and spectral information) of the tissue surface [46] 39. Adaptive neuro-fuzzy inference system for surgical use [47] 40. Laparoscopic skills assessment [48] 41. Measurement of the hand motions of expert and novice surgeons [49] 42. Passive magnetic tracking for untethered medical instruments [50] 43. Real-time tissue deformation in image-guided neurosurgery [51] 44. Prediction of different outcome variables of PCNL [52] 45. Predictive control of semi-active Prosthetic Knee [53] |
| 3 | **Autoencoder** | 1. 3D point cloud reconstruction from single monocular endoscopic images [54] 2. Renal-CAD for assessing kidney transplant function [55] 3. Online automated and efficient surgical skills assessment technique [56] 4. Automatic staging of distal femur trochlear dysplasia [57] |
| 4 | **Classiﬁcation** | 1. Multi-task multi-stage temporal convolutional network for surgical activity recognition on LVSG dataset [58] 2. Automated Classification of IONM [59] 3. Detection of procedural knowledge in robotic-assisted surgical texts [60] 4. Particle Filter Object Tracking [61] 5. AI CC Diagnosis Using Clinical Data [5] 6. Cross-modal learning for gesture and skill recognition in RAS [62] 7. NLP surgical phase recognition in LC [63] 8. Decision-Making for CC Cancer Treatment Strategy [64] 9. Surgeons’ subjective skill levels prediction via NIRS [65] 10. LTHRM Big Data Functional Analysis [66] 11. AIRAM in colorectal surgery [67] 12. Intraoperative safety [11] 13. Performance metrics during GS-SP predict surgeon experience in VUA [1] 14. Classification of GB wall vascularity [68] 15. TEF for MIS in the Hepatoduodenal Area [2] 16. A NLBPM for SLNS in FESS [69] 17. LSTM in Time-series data from Basic Laparoscopic Urologic Skills dataset [70] 18. Visual gaze patterns to identify risk of bile duct injury during LC [71] 19. Computer vision to recognize and classify suturing gestures in RAS [72] 20. Automatic liver graft segmentation [73, 74] 21. Automated workflow recognition in LCRS [22] 22. Pancreas segmentation and station recognition by a EUS training tool [75] 23. Intra-abdominal acoustic feedback improves the perception during MIS [76] 24. Intraoperative Localization of STN During DBS Surgery [77] 25. Automatic selection of informative laryngoscopic-video frames [78, 79] 26. Automated ultrasound CAD of thyroid lesions in surgical office [80] 27. Prediction of surgical site infection [26] 28. Representation of instrument images in laparoscopy videos [81] 29. Online automated and efficient surgical skills assessment technique [56] 30. Algorithm for Gleason Grading of Prostate Cancer Biopsies [82] 31. Multi-instance multi-label annotation for LC images [83] 32. Eye-tracking metrics and perceived workload in RAS [84] 33. DRS of human joint tissue [85] 34. Force classification during robotic interventions [34] 35. Vessel pattern characterization of larynx via CE + NBI images [86] 36. Future-State Predicting LSTM for Early Surgery Type Recognition [87] 37. A DSS for large kidney stone treatment [88] 38. Classification and analysis of surgical skill levels [89] 39. PAWSS for visual tracking in surgical video [90] 40. Resectability prediction of HIPEC [91] 41. Video-based surgical skill assessment [92] 42. Classification of FNAC breast images [93] 43. HIS for tissue classification in LCRS [94] 44. Invasion depth of the GC based on conventional endoscopy [41] 45. Laparoscopic skills classification using Apple watch motion signals [42] 46. Surgical SATR-DL and Task Recognition In RAS [95] 47. HMI for intuitive vision control and continuous surgical operation [96] 48. A Neuromuscular Interface for Robotic Devices Control [45] 49. Radiomics nomogram prediction of the malignant potential of GISTs [97] 50. pCLE brain tumor characterization [98] 51. Objective Assessment of Surgical Psychomotor Skills via an open source Laparoscopic Box Trainer [48] 52. Functional Brain States Measure Mentor-Trainee Trust during RAS [99] 53. Endoscopic Image Classification via Clustered Convolutional Features [100] 54. Automated skills assessment of laparoscopic training tasks [101] 55. Energy-Based Metrics for Arthroscopic Skills Assessment [102] 56. Automatic detection of surgical hemorrhage using computer vision [103] 57. Automated robot-assisted surgical skill evaluation: Predictive analytics approach [104] 58. Predicting surgical skill from the first N seconds of a task: value over task time using the isogony principle [105] 59. Addressing multi-label imbalance problem of surgical tool detection using CNN [106] |
| 5 | **Clustering** | 1. Cross-modal self-supervised representation learning for gesture and skill recognition in RAS [62] 2. LTHRM Big Data Functional Analysis [66] 3. Application of Advanced Bioinformatics to Understand and Predict Burnout Among Surgical Trainees [107] 4. Towards near real-time assessment of surgical skills: A comparison of feature extraction techniques [56] 5. Multi-instance multi-label annotation for LC images [83] 6. Predicting the quality of surgical exposure using spatial and procedural features from laparoscopic videos [108] 7. Virtual pointer for gaze guidance in LS [109] 8. Accurate and interpretable evaluation of surgical skills from kinematic data [110] 9. Tactile sensor-based real-time clustering for tissue differentiation [111] 10. Deeply-learnt damped least-squares (DL-DLS) method for inverse kinematics of snake-like robots [112] 11. Endoscopic Image Classification and Retrieval using Clustered Convolutional Features [100] 12. Predicting surgical skill from the first N seconds of a task: value over task time using the isogony principle [105] |
| 6 | **Convolutional neural networks (CNNs)** | 1. Operating hands detection in open surgery videos [113] 2. Video Archiving and Communication System (VACS [4]) 3. Automated Classification of IONM [59] 4. Multi-task multi-stage temporal convolutional network for surgical activity recognition, on a GB dataset [58] 5. Addressing multi-label imbalance problem of surgical tool detection using CNN [106] 6. Real time colorectal polyps video detection on a single GPU [114] 7. A Wearable System to Avoid Face Touching [115] 8. Automatic tip detection of surgical instruments in BESS [116] 9. Automatic Segmentation of Oral and Oropharyngeal Cancer Using NBI [117] 10. Nerve recognition in transforaminal endoscopic discectomy [118] 11. Automate surgical skill assessment in LC videos [119] 12. Detection, segmentation, and 3D pose estimation of surgical tools [120] 13. Automated Vision-Based Microsurgical Skill Analysis in Neurosurgery [12] 14. See-Through Vision with Unsupervised Scene Occlusion Reconstruction [121] 15. A contextual detector of surgical tools in laparoscopic videos [122] 16. CT based preoperative diagnosis of occult peritoneal metastasis in advanced GC [123] 17. Classification of GB wall vascularity [68] 18. Intraoperative margin assessment in oral and oropharyngeal cancer using FLIm [124] 19. Defining the margins of early GC using magnifying NBI endoscopy [125] 20. Automated operative phase identification using POEM [21] 21. Multi-scale attention single shot detector for surgical instruments [126] 22. Automated and real-time validation of gastroesophageal varices under esophagogastroduodenoscopy [127] 23. LRTD based active learning for surgical workflow recognition [128] 24. Automated LCRS workflow recognition [22] 25. Detecting the occluding contours of the uterus to automatize augmented laparoscopy [129] 26. TTTS Inter-fetus membrane segmentation (n=3) [31, 130, 131] 27. MLFA-Net for identifying surgical instruments in endoscopic images [132] 28. CNN-based surgical instrument detection [133] 29. Intra-abdominal acoustic feedback improves the perception during MIS [76] 30. Learned optical flow for intra-operative tracking of the retinal fundus [134] 31. Automatic selection of informative laryngoscopic-video frames [78] 32. Automated Surgical Instrument Detection from Laparoscopic Gastrectomy Video Images [135] 33. Retrospective imaging studies of GC [25] 34. Automatic 3D landmarking model for CT image of oral and maxillofacial surgery [136] 35. Automatic gauze tracking in laparoscopic surgery using image texture analysis [137] 36. Real-time surgical instrument detection in RAS (n=2) [27, 81] 37. Deep learning for computerized laparoscopic image understanding in gynecology [138] 38. A Modified Encoder-Decoder U-Net Architecture for Semantic and Instance Segmentation of Surgical Instruments from Laparoscopic Images [139] 39. encoder-decoder siamese network for knee cartilage tracking in ultrasound images [140] 40. 3D posture estimation of robot forceps using endoscope [141] 41. Real-time automatic surgical phase recognition in laparoscopic sigmoidectomy [142] 42. Towards near real-time assessment of surgical skills: A comparison of feature extraction techniques [56] 43. Assessment of bowel preparation [32] 44. Algorithm for Gleason Grading of Prostate Cancer Biopsies [82] 45. Generative Cooperative Networks for Joint Surgical Smoke Detection [143] 46. Detection and segmentation of multi-class artifacts in endoscopy and Removal [144] 47. Multi-task recurrent convolutional network with correlation loss for surgical video analysis [145] 48. AR navigation for liver resection with a stereoscopic laparoscope [146] 49. Accurate and interpretable evaluation of surgical skills from kinematic data [110] 50. Future-State Predicting LSTM for Early Surgery Type Recognition [87] 51. Novel evaluation of surgical activity recognition models using task-based efficiency metrics [147] 52. A CNN-based prototype method of unstructured surgical state perception and navigation for an endovascular surgery robot [148] 53. EasyLabels: weak labels for scene segmentation in laparoscopic videos [149] 54. Video-based surgical skill assessment using 3D convolutional neural networks [92] 55. A tool to detect anatomical landmarks in X-ray images independent of their viewing direction [150] 56. Weakly supervised convolutional LSTM approach for tool tracking in laparoscopic videos [151] 57. Prediction of laparoscopic procedure duration using unlabeled, multimodal sensor data [152] 58. Classification of FNAC breast images [93] 59. Endoscopic detection of early GC without blind spots [153] 60. Real-time tracking of surgical instruments based on spatio-temporal context and deep learning [154] 61. A dataset of laryngeal endoscopic images with comparative study on convolution neural network-based semantic segmentation [155] 62. Invasion depth of the GC based on conventional endoscopy [41] 63. Objective skill evaluation in RAS [156] 64. Dual-modality endoscopic probe for tissue surface shape reconstruction and hyperspectral imaging enabled by deep neural networks [46] 65. A deep learning approach for real time prostate segmentation in freehand ultrasound guided biopsy [157] 66. Articulated Multi-Instrument 2-D Pose Estimation Using Fully Convolutional Networks [158] 67. A deep learning approach for pose estimation from volumetric OCT data [159] 68. Endoscopic Image Classification via Clustered Convolutional Features [100] 69. Surgical-tools detection based on Convolutional Neural Network in laparoscopic robot-assisted surgery [160] 70. Tracking of surgical instruments in minimally invasive surgery via the convolutional neural network deep learning-based method [161] 71. Detection and Localization of Robotic Tools in RAS Videos Using Deep Neural Networks for Region Proposal and Detection [162] 72. EndoNet: A Deep Architecture for Recognition Tasks on Laparoscopic Videos [163] |
| 7 | **Decision trees** | 1. Factors That Predict Sagittal Plane Knee Biomechanical Symmetry After Anterior Cruciate Ligament Reconstruction: A Decision Tree Analysis [164] 2. Early-stage non-small cell lung cancer beyond life expectancy: Still not too old for surgery? [165] 3. Robotic Ureteral Reconstruction [166] 4. Cost-effectiveness of Surgical Treatment Pathways for Prolapse [167] 5. European Association of Urology Guidelines on Muscle-invasive and Metastatic Bladder Cancer: Summary of the 2020 Guidelines [168] 6. Comparative cost of transoral robotic surgery and radiotherapy (IMRT) in early stage tonsil cancer [169] 7. Prospective Implementation and Evaluation of a Decision-Tree Algorithm for Route of Hysterectomy [170] 8. Using Surgeon Hand Motions to Predict Surgical Maneuvers [171] 9. Predicting acute kidney injury after robot-assisted partial nephrectomy: Implications for patient selection and postoperative management [172] 10. Cost-effectiveness analysis of robot-assisted vs. open partial nephrectomy [173] 11. Development of a Patient-Based Model for Estimating Operative Times for RAPR [174] 12. Development of a patient and institutional-based model for estimation of operative times for robot-assisted radical cystectomy: results from the International Robotic Cystectomy Consortium [175] 13. Determining Optimal Route of Hysterectomy for Benign Indications: Clinical Decision Tree Algorithm [176] 14. Cost-effectiveness of robot-assisted partial nephrectomy for the prevention of perioperative complications [177] 15. Making a case for high-volume robotic surgery centers: A cost-effectiveness analysis of transoral robotic surgery [178] 16. Robotic versus laparoscopic Roux-en-Y gastric bypass (RYGB) in obese adults ages 18 to 65 years: a systematic review and economic analysis [179] 17. Robot-assisted radical cystectomy versus open radical cystectomy: a complete cost analysis [180] |
| 8 | **Dimensionality reduction** | 1. Development of nomogram models of inflammatory markers based on clinical database to predict prognosis for hepatocellular carcinoma after surgical resection [181] 2. Contribution of revision amputation vs replantation for certain digits to functional outcomes after traumatic digit amputations: A comparative study based on multicenter prospective cohort [182] 3. Predicting kidney graft survival using machine learning methods: Prediction model development and feature significance analysis study [183] 4. Applying a random projection algorithm to optimize machine learning model for predicting peritoneal metastasis in GC patients using CT images [184] 5. A rule-based semantic approach for data integration, standardization and dimensionality reduction utilizing the UMLS: Application to predicting bariatric surgery outcomes [185] 6. Modeling and segmentation of surgical workflow from laparoscopic video [186] |
| 9 | **Dynamic time warping (DTW)** | 1. Validation of virtual reality arthroscopy simulator relevance in characterising experienced surgeons [187] 2. Offline identification of surgical deviations in laparoscopic rectopexy [188] 3. Sensor-based machine learning for workflow detection and as key to detect expert level in laparoscopic suturing and knot-tying [39] 4. An automatic skill evaluation framework for RS training [189] 5. Evaluation of RS skills using dynamic time warping [190] 6. Automatic matching of surgeries to predict surgeons' next actions [191] 7. Classification of surgical processes using dynamic time warping [192] |
| 10 | **Ensemble learning** | 1. Efficient Reject Options for Particle Filter Object Tracking in Medical Applications [61] 2. Preoperatively predict immediate remission after TSS in CD patients [193] 3. Data analytics interrogates robotic surgical performance using a microsurgery-specific haptic device [194] 4. Retrospective PM detection in GC [25] 5. Prediction of surgical site infection [26] 6. Assessment of peritoneal microbial features and tumor marker levels as potential diagnostic tools for ovarian cancer [195] 7. Real-time monitoring radiofrequency ablation using tree-based ensemble learning models [3] 8. Workflow detection and of expert level in laparoscopic suturing and knot-tying [39] 9. A dataset of laryngeal endoscopic images with comparative study on convolution neural network-based semantic segmentation [155] 10. "Deep-Onto" network for surgical workflow and context recognition [196] 11. Surgical SATR-DL and NBI Task Recognition in RAS [95] |
| 11 | **Feed forward neural network** | 1. A Bayesian finite-element trained machine learning approach for predicting post-burn contraction [197] 2. Deep Neural Networks Outperform the CAPRA Score in Predicting Biochemical Recurrence After Prostatectomy [198] 3. Temporal Lobe Epilepsy Surgical Outcomes Can Be Inferred Based on Structural Connectome Hubs: A Machine Learning Study [199] 4. Computer Aided Diagnosis for Confocal Laser Endomicroscopy in Advanced Colorectal Adenocarcinoma [200] |
| 12 | **Fully convolutional networks** | 1. Automatic Segmentation of Oral and Oropharyngeal Cancer Using NBI [117] 2. Detection, segmentation, and 3D pose estimation of surgical tools using CNN and algebraic geometry [120] 3. See-Through Vision with Unsupervised Scene Occlusion Reconstruction [121] 4. Detecting the occluding contours of the uterus to automatize augmented laparoscopy [129] 5. Intra-abdominal acoustic feedback improves the perception during MIS [76] 6. Weakly supervised convolutional LSTM approach for tool tracking in laparoscopic videos [201] 7. TTTS Inter-fetus membrane segmentation [31] 8. Accurate and interpretable evaluation of surgical skills from kinematic data using FCNNs [110] 9. EasyLabels: weak labels for scene segmentation in laparoscopic videos [149] 10. Articulated Multi-Instrument 2-D Pose Estimation Using FCNN [158] 11. Addressing multi-label imbalance problem of surgical tool detection using CNN [106] |
| 13 | **Gated recurrent units (GRUs)** | 1. Segmenting and classifying activities in robot-assisted surgery with recurrent neural networks [202] |
| 14 | **Generative Adversarial Networks (GAN)** | 1. See-Through Vision with Unsupervised Scene Occlusion Reconstruction [121] 2. Towards realistic laparoscopic image generation using image-domain translation [18] 3. Using conditional GANs to reduce the effects of latency in robotic telesurgery [203] 4. Combination of GAN and CNN for automatic subcentimeter pulmonary adenocarcinoma classification [204] 5. Optimization of virtual and real registration technology based on AR in a surgical navigation system [205] 6. Generative Cooperative Networks for Joint Surgical Smoke Detection and Removal [143] 7. Unsupervised Reverse Domain Adaptation for Synthetic Medical Images via Adversarial Training [206] |
| 15 | **Gradient boosting** | 1. Prediction of surgeon’s experience [1, 207] |
| 16 | **Hidden Markov models (HMMs)** | 1. NLP surgical phase recognition in LC [63] 2. A Gesture Recognition Algorithm for Hand-Assisted LS [208] 3. Segmenting and classifying activities in RAS with RNN [202] |
| 17 | **Imitation learning** | 1. Towards Autonomous Eye Surgery by Combining Deep Imitation Learning with Optimal Control [209] 2. Application of artificial intelligence in surgery [210] 3. Motion2Vec: semi-supervised representation learning from surgical videos [211] |
| 18 | **Instance segmentation** | 1. Vessel segmentation for automatic registration of untracked laparoscopic ultrasound to CT of the liver [212] 2. Heidelberg colorectal data set for surgical data science in the sensor operating room [213] 3. TTTS Inter-fetus membrane segmentation [214] 4. Detection, segmentation, and 3D pose estimation of surgical tools using CNN and algebraic geometry [120] 5. Towards realistic laparoscopic image generation using image-domain translation [18] 6. A Modified Encoder-Decoder U-Net Architecture for Semantic and Instance Segmentation of Surgical Instruments from Laparoscopic Images [139] 7. Kidney edge detection in laparoscopic image data for computer-assisted surgery [215] 8. Multi-instance multi-label annotation for LC images [83] 9. Combining Differential Kinematics and Optical Flow for Automatic Labeling of Continuum Robots in MIS [216] 10. A Platform Integrating Acquisition, Reconstruction, Visualization, and Manipulator Control Modules for MRI-Guided Interventions [217] |
| 19 | **JHU-ISI Gesture and Skill Assessment Working Set (JIGSAWS) dataset** | 1. Multi-task multi-stage temporal convolutional network for surgical activity recognition on LVSG dataset [58] 2. Surgical Hand Gesture Recognition Utilizing Electroencephalogram as Input to the Machine Learning and Network Neuroscience Algorithms [218] 3. Cross-modal learning for gesture and skill recognition in robotic surgery [62] 4. Computer vision to recognize and classify suturing gestures in RAS [72] 5. Transrectal ultrasound image-based real-time augmented reality guidance in robot-assisted LCRS: a proof-of-concept study [219] 6. A Gesture Recognition Algorithm for Hand-Assisted LS [208] 7. Virtual pointer for gaze guidance in LS [109] 8. A virtual pointer to support the adoption of professional vision in laparoscopic training [220] 9. Accurate and interpretable evaluation of surgical skills from kinematic data using fully convolutional neural networks [110] 10. Online automated and efficient surgical skills assessment technique [56] 11. Classification and analysis of surgical skill levels [89] 12. Video-based surgical skill assessment [92] 13. Segmenting and classifying activities in RAS with RNN [202] 14. Surgical SATR-DL and Task Recognition In RAS [95] 15. Objective skill evaluation in RAS [156] 16. Design and evaluation of an eye tracking support system for the scrub nurse [221] 17. Gesture segmentation and classification using affine speed and energy [222] 18. Detection and Localization of Robotic Tools in RAS Videos Using Deep Neural Networks for Region Proposal and Detection [162] |
| 20 | ***k*-nearest neighbors (*k*NN)** | 1. Minimally Invasive Optically Guided Surgery [2] 2. Breakthrough Detection Method for Bone Drilling [30] 3. Automated robot-assisted surgical skill evaluation: Predictive analytics approach [104] |
| 21 | **Kernel** | 1. Adaptive kernel selection network with attention constraint for surgical instrument classification [223] 2. Analysis of the Effect of Robots in the Treatment of Pancreatic Cancer Based on Smart Medicine [224] 3. Properties of the EQ-5D-5L when prospective longitudinal data from 28,902 total hip arthroplasty procedures are applied to different European EQ-5D-5L value sets [225] 4. Adverse events reporting in stage III NSCLC trials investigating surgery and radiotherapy [226] 5. Splenic Infarction as a Delayed Febrile Complication Following Radical Gastrectomy for GC Patients: Computed Tomography-Based Analysis [227] 6. Predicting colorectal surgical complications using heterogeneous clinical data and kernel methods [228] 7. A systematic approach to predicting the risk of unicompartmental knee arthroplasty revision [229] |
| 22 | **Lasso regression** | 1. Using Nomograms to Predict the PPCs of Patients With Diffuse Peritonitis Undergoing Emergency Gastrointestinal Surgery [230] 2. Development and validation of a machine learning-based nomogram for prediction of intrahepatic cholangiocarcinoma in patients with intrahepatic lithiasis [231] 3. Development of a prediction model based on LASSO regression to evaluate the risk of non-sentinel lymph node metastasis in Chinese breast cancer patients with 1-2 positive sentinel lymph nodes [232] 4. LASSO-Based Machine Learning Algorithm for Prediction of Lymph Node Metastasis in T1 CC [233] 5. Predictive nomogram for postoperative pancreatic fistula following pancreaticoduodenectomy: a retrospective study [234] 6. Artificial neural networks versus LASSO regression for the prediction of long-term survival after surgery for invasive IPMN of the pancreas [235] 7. Risk factors and socio-economic burden in pancreatic ductal adenocarcinoma operation: a machine learning based analysis [236] 8. Prognostic study for survival outcome following the treatment of second primary lung cancer in patients with previously resected non-small cell lung cancer [237] 9. Integrated nomograms to predict overall survival and recurrence-free survival in patients with combined hepatocellular cholangiocarcinoma (cHCC) after liver resection [238] 10. Using machine learning to construct nomograms for patients with metastatic colon cancer [239] 11. Modified Colon Leakage Score to Predict Anastomotic Leakage in Patients Who Underwent Left-Sided Colorectal Surgery [240] 12. Evaluating and Predicting the Probability of Death in Patients with Non-Metastatic Osteosarcoma: A Population-Based Study [241] 13. Development and Validation of a Nomogram for Preoperative Prediction of Perineural Invasion in CC [242] 14. A Machine Learning Approach to Predicting Case Duration for Robot-Assisted Surgery [243] |
| 23 | **Logistic regression** | 1. Automated robot-assisted surgical skill evaluation: Predictive analytics approach [104] 2. Predictors of 30-Day Mortality Among Dutch Patients Undergoing CC Surgery, 2011-2016 [207] 3. CVS and intraoperative events during LC are associated with disease severity [6] 4. Preoperatively predict immediate remission after TSS in CD patients [193] 5. CT based preoperative diagnosis of occult peritoneal metastasis in advanced GC [123] 6. Analyzing National Incidences and Predictors of Open Conversion During MIPN for cT1 Renal Masses [244] 7. Predicting intra-operative and postoperative consequential events using machine-learning techniques in patients undergoing robot-assisted partial nephrectomy: a Vattikuti Collective Quality Initiative database study [245] 8. Marginal radiomics features as imaging biomarkers for pathological invasion in lung adenocarcinoma[246] 9. Development and external validation of a nomogram to predict lymph node invasion after robot assisted radical prostatectomy [247] 10. Morbidity and mortality prediction after LVSG [33] 11. Salivary metabolomics with alternative decision tree-based machine learning methods for breast cancer discrimination [248] 12. Predictors of blood transfusion use in robotic beating-heart totally endoscopic coronary artery bypass with anastomotic connectors [249] 13. A preoperative nomogram to predict major complications after robot assisted partial nephrectomy [250] 14. Using Machine Learning Applied to Real-World Healthcare Data for Predictive Analytics: An Applied Example in Bariatric Surgery [251] 15. A computer vision technique for automated assessment of surgical performance using surgeons' console-feed videos [252] 16. Machine learning to predict lung nodule biopsy method using CT image features: A pilot study [253] 17. Use of machine learning to predict early biochemical recurrence after robot-assisted prostatectomy [254] |
| 24 | **Long short-term memory (LSTM)** | 1. Multi-task multi-stage temporal convolutional network for surgical activity recognition, on a GB dataset [58] 2. Automated Classification of IONM [59] 3. LSTM in Time-series data from Basic Laparoscopic Urologic Skills dataset [70] 4. Future-State Predicting LSTM for Early Surgery Type Recognition [87] 5. Novel evaluation of surgical activity recognition models using task-based efficiency metrics [147] 6. Weakly supervised convolutional LSTM approach for tool tracking in laparoscopic videos [151] 7. Segmenting and classifying activities in RAS with RNN [202] 8. Deep Learning-Based Haptic Guidance for Surgical Skills Transfer [255] 9. End-Effector Contact and Force Detection for Miniature Autonomous Robots Performing Lunar and Expeditionary Surgery [256] 10. Vision-Based Suture Tensile Force [15] 11. Language-based translation and prediction of surgical navigation steps for endoscopic wayfinding assistance in MIS [69] 12. Computer vision to recognize and classify suturing gestures in RAS [72] 13. Automated computer vision in POEM [21] 14. FetNet: a recurrent convolutional network for occlusion identification in tttS fetoscopic videos [131] 15. State recognition of decompressive laminectomy with multiple information in RAS [257] 16. Multi-task recurrent convolutional network with correlation loss for surgical video analysis [145] 17. Towards Retrieving Force Feedback in Robotic-Assisted Surgery: A Supervised Neuro-Recurrent-Vision Approach [258] |
| 26 | **Multilayer perceptrons** | 1. Machine Learning and Feature Selection Applied to SEER Data to Reliably Assess Thyroid Cancer Prognosis [259] 2. Overview of Deep Learning in Gastrointestinal Endoscopy [260] 3. Prediction of postoperative prostatic cancer stage on the basis of systematic biopsies using two types of artificial neural networks [261] |
| 27 | **Object detection** | 1. Operating hands detection in open surgery videos [113] 2. Laparoscopic system for simultaneous high-resolution video and rapid hyperspectral imaging in the visible and near-infrared spectral range [262] 3. Multi-scale attention single shot detector for surgical instruments [126] 4. Object extraction via deep learning-based marker-free tracking framework of surgical instruments for laparoscope-holder robots [263] 5. Detecting the occluding contours of the uterus to automatize augmented laparoscopy [129] 6. Multi-level feature aggregation network for instrument identification of endoscopic images [132] 7. Development of an artificial intelligence system using deep learning to indicate anatomical landmarks during LC [264] 8. Real-time surgical instrument detection in RAS [27] 9. Representation of instrument images in laparoscopy videos [81] 10. PAWSS for visual tracking in surgical video[90] 11. EasyLabels: weak labels for scene segmentation in laparoscopic videos [149] 12. Keyframe extraction from laparoscopic videos based on visual saliency detection [265] 13. Long Term Safety Area Tracking (LT-SAT) with online failure detection and recovery for robotic minimally invasive surgery [266] 14. Detection and Localization of Robotic Tools in RAS Videos Using Deep Neural Networks for Region Proposal and Detection [162] |
| 28 | **Principal component analysis** | 1. A Prognostic Model Using Immune-Related Genes for CC [267] 2. Raman hyperspectral imaging coupled to three-dimensional discriminant analysis: Classification of meningiomas brain tumour grades [268] 3. Colonoscope retraction technique and predicting adenoma detection rate: a multicenter study [269] 4. Survival stratification for CC via multi-omics integration using an autoencoder-based model [270] 5. Detection of pancreatic cancer by convolutional-neural-network-assisted spontaneous Raman spectroscopy with critical feature visualization [271] 6. Identification of the immune cell infiltration landscape in pancreatic cancer to assist immunotherapy [272] 7. Principal Component Analysis of Knee Joint Differences Between Bilateral and Unilateral Total Knee Replacement Patients During Level Walking [273] 8. Motion analysis for better understanding of psychomotor skills in laparoscopy: objective assessment-based simulation training using animal organs [274] 9. Scaphoid fracture displacement is not correlated with the fracture angle [275] 10. Differences in intestinal microbiota profiling after upper and lower gastrointestinal surgery [276] 11. Survey to define informational needs of patients undergoing surgery for Crohn's anal fistula [277]. 12. Objective assessment of obstetrics residents' surgical skills in caesarean: Development and evaluation of a specific rating scale [278] 13. How we assess the perioperative anxiety of surgical patients with pulmonary nodules: the revision of state-trait anxiety inventory [279] 14. A machine learning approach identified a diagnostic model for pancreatic cancer through using circulating microRNA signatures [280] 15. Automatic detection of symmetry plane for computer-aided surgical simulation in craniomaxillofacial surgery [281] 16. Application of Advanced Bioinformatics to Understand and Predict Burnout Among Surgical Trainees [107] 17. Towards near real-time assessment of surgical skills: A comparison of feature extraction techniques [56] 18. Three-dimensional hernia analysis: the impact of size on surgical outcome [282] 19. Scaphoid Fracture Patterns-Part One: Three-Dimensional Computed Tomography Analysis [283] 20. Raman Spectroscopy for Rapid Evaluation of Surgical Margins during Breast Cancer Lumpectomy [284] 21. Raman Spectroscopy Analysis for Optical Diagnosis of Oral Cancer Detection [285] 22. The long term microbiota and metabolic status in patients with CC after curative colon surgery [286] 23. Discrimination of cancerous and healthy colon tissues: A new laser-based method [287] 24. The Colonoscopy Satisfaction and Safety Questionnaire (CSSQP) for CC Screening: A Development and Validation Study [288] 25. Breast Shape Analysis With Curvature Estimates and Principal Component Analysis for Cosmetic and Reconstructive Breast Surgery [289] 26. Association of Expectations of Training With Attrition in General Surgery Residents [290] 27. Interpretation of motion analysis of laparoscopic instruments based on principal component analysis in box trainer settings [291] 28. The surgical intelligent knife distinguishes normal, borderline and malignant gynaecological tissues using rapid evaporative ionisation mass spectrometry (REIMS) [292] 29. Development of an instrument evaluating the impact of surgeon-patient relationship in patients on sick leave [293] |
| 29 | **Random Forests** | 1. Prediction of surgeon’s experience [1] 2. Predictors of 30-Day Mortality Among Dutch Patients Undergoing CC Surgery, 2011-2016 [207] 3. Intraoperative margin assessment in oral and oropharyngeal cancer using FLIm [124] 4. Risk assessment for intra-abdominal injury following blunt trauma in children: Derivation and validation of a machine learning model [294] 5. Predicting intra-operative and postoperative consequential events using machine-learning techniques in patients undergoing robot-assisted partial nephrectomy: a Vattikuti Collective Quality Initiative database study [245] 6. Preoperative CT-based radiomics combined with intraoperative frozen section is predictive of invasive adenocarcinoma in pulmonary nodules: a multicenter study [295] 7. A novel automated lumen segmentation and classification algorithm for detection of irregular protrusion after stents deployment [296] 8. Vessel pattern characterization of larynx via CE + NBI images [86] 9. Resectability prediction of HIPEC [91] 10. Using Surgeon Hand Motions to Predict Surgical Maneuvers [171] 11. Monitoring radiofrequency ablation depth [3] 12. A Machine Learning Approach to Predicting Case Duration for RAS [243] 13. Machine learning to predict lung nodule biopsy method using CT image features: A pilot study [253] 14. Use of machine learning to predict early biochemical recurrence after robot-assisted prostatectomy [254] 15. Utilizing Machine Learning and Automated Performance Metrics to Evaluate Robot-Assisted Radical Prostatectomy Performance and Predict Outcomes [297] |
| 30 | **Recurrent neural networks (RNNs)** | 1. Segmenting and classifying activities in RAS with RNN [202] 2. A contextual detector of surgical tools in laparoscopic videos [122] 3. Improved recurrent neural network-based manipulator control with remote center of motion constraints: Experimental results [298] 4. LRTD based active learning for surgical workflow recognition [128] 5. FetNet: a recurrent convolutional network for occlusion identification in tttS fetoscopic videos [131] 6. Multi-task recurrent convolutional network with correlation loss for surgical video analysis [145] 7. Developed and validated a prognostic nomogram for recurrence-free survival after complete surgical resection of local primary GIST based on deep learning [299] 8. A deep learning approach for real time prostate segmentation in freehand ultrasound guided biopsy [157] 9. Towards Retrieving Force Feedback in RAS: A Supervised Neuro-Recurrent-Vision Approach [258] |
| 31 | **Regression** | 1. Particle Filter Object Tracking [61] 2. Predictors of 30-Day Mortality Among Dutch Patients Undergoing CC, 2011-2016 [207] 3. AI CC Diagnosis Using Clinical Data [5] 4. CVS and intraoperative events during LC are associated with disease severity [6] 5. Data-Driven Shape Sensing of a Surgical Continuum Manipulator Using an Uncalibrated Fiber Bragg Grating Sensor [300] 6. Automate surgical skill assessment in LC videos [119] 7. CT based preoperative diagnosis of occult peritoneal metastasis in advanced GC [123] 8. Spontaneous pregnancy rate following surgery for deep infiltrating endometriosis in infertile women: The impact of the learning curve [301] 9. Three Different Learning Curves Have an Independent Impact on Perioperative Outcomes After Robotic Partial Nephrectomy: A Comparative Analysis [302] 10. Data analytics interrogates robotic surgical performance using a microsurgery-specific haptic device [194] 11. CT Fluoroscopy Guided Thoracic Biopsies (CTTB) Are Highly Accurate and Safe: Outcomes and Predictive Modeling of Complications Utilizing Machine Learning [303] 12. Analyzing National Incidences and Predictors of Open Conversion During MIPN for cT1 Renal Masses [244] 13. Multi-level feature aggregation network for instrument identification of endoscopic images [132] 14. Predicting intra-operative and postoperative consequential events using machine-learning techniques in patients undergoing robot-assisted partial nephrectomy: a Vattikuti Collective Quality Initiative database study [245] 15. Contemporary Rates and Predictors of Open Conversion During Minimally Invasive Radical Prostatectomy for Nonmetastatic Prostate Cancer [304] 16. Prediction of surgical site infection [26] 17. Real-time surgical instrument detection in RAS [27] 18. Retrospective Cohort Analysis from a High-Volume Center of Prognostic Factors Affecting Biochemical Relapse in Patients with Encapsulated, Margin-Negative, Isolated Seminal Vesicle Invasion After Robot-Assisted Laparoscopic Prostatectomy: A Novel Study [305] 19. Development and external validation of a nomogram to predict lymph node invasion after robot assisted radical prostatectomy [247] 20. Morbidity and mortality prediction after LVSG [33] 21. Accurate and interpretable evaluation of surgical skills from kinematic data using fully convolutional neural networks [110] 22. Salivary metabolomics with alternative decision tree-based machine learning methods for breast cancer discrimination [248] 23. Predictors of blood transfusion use in robotic beating-heart totally endoscopic coronary artery bypass with anastomotic connectors [249] 24. A preoperative nomogram to predict major complications after robot assisted partial nephrectomy [250] 25. Using Machine Learning Applied to Real-World Healthcare Data for Predictive Analytics: An Applied Example in Bariatric Surgery [251] 26. Quantified pre-operative neurological dysfunction predicts outcome after coronary artery bypass surgery [306] 27. Current Management of pT3b Prostate Cancer After Robot-assisted Laparoscopic Prostatectomy [307] 28. Workflow detection and of expert level in laparoscopic suturing and knot-tying [39] 29. A Machine Learning Approach to Predicting Case Duration for RAS [243] 30. A computer vision technique for automated assessment of surgical performance using surgeons' console-feed videos [252] 31. Machine learning to predict lung nodule biopsy method using CT image features: A pilot study [253] 32. Estimation of grip force and jaw angle on a da Vinci surgical tool using back-end sensors alone [43] 33. Real and predicted mortality under health spending constraints in Italy [44] 34. Radiomics nomogram prediction of the malignant potential of GISTs [97] 35. Use of machine learning to predict early biochemical recurrence after robot-assisted prostatectomy [254] 36. Articulated Multi-Instrument 2-D Pose Estimation Using Fully Convolutional Networks [158] 37. A deep learning approach for pose estimation from volumetric OCT data [159] 38. Automatically rating trainee skill at a pediatric laparoscopic suturing task [308] 39. Natural History and Predictors of Parastomal Hernia after Robot-Assisted Radical Cystectomy and Ileal Conduit Urinary Diversion [309] 40. Real-time tissue deformation in image-guided neurosurgery [51] 41. Robot-assisted surgery in a broader healthcare perspective: a difference-in-difference-based cost analysis of a national prostatectomy cohort [310] 42. Anticipation, teamwork and cognitive load: chasing efficiency during RAS [311] 43. Automated robot-assisted surgical skill evaluation: Predictive analytics approach [104] 44. Using Contact Forces and Robot Arm Accelerations to Automatically Rate Surgeon Skill at Peg Transfer [312] 45. A comparison of trends in operative approach and postoperative outcomes for CC surgery [313] 46. Data-driven methods towards learning the highly nonlinear inverse kinematics of tendon-driven surgical manipulators [314] 47. The combined effects of action observation and passive proprioceptive training on adaptive motor learning [315] 48. Tumor diameter accurately predicts perioperative outcomes in T1 renal cancer treated with robot-assisted partial nephrectomy [316] 49. Impact of novel techniques on minimally invasive adrenal surgery: trends and outcomes from a contemporary international large series in urology [317] |
| 32 | **Reinforcement learning** | 1. What Are the Tradeoffs in Outcomes after Casting Versus Surgery for Closed Extraarticular Distal Radius Fractures in Older Patients? A Statistical Learning Model [318] 2. Evaluation of the effects of an artificial intelligence system on endoscopy quality and preliminary testing of its performance in detecting early GC: a randomized controlled trial [319] 3. Machine learning in gastrointestinal surgery [320] 4. Machine Learning and Artificial Intelligence for Surgical Decision Making [321] 5. Machine learning: principles and applications for thoracic surgery [322] 6. Reinforcement learning in surgery [323] 7. Artificial intelligence in perioperative management of major gastrointestinal surgeries [324] 8. Artificial Intelligence in Plastic Surgery: Applications and Challenges [325] 9. Machine learning in the optimization of robotics in the operative field [326] 10. Opportunities for machine learning to improve surgical ward safety [327] 11. Decision analysis and reinforcement learning in surgical decision-making [328] 12. Variable Admittance Control Based on Fuzzy Reinforcement Learning for Minimally Invasive Surgery Manipulator [329] 13. Development of a colon endoscope robot that adjusts its locomotion through the use of reinforcement learning [330] |
| 33 | **Representational learning** | 1. Recovering dense 3D point clouds from single endoscopic image [54] 2. Cross-modal self-supervised representation learning for gesture and skill recognition in RAS [62] 3. Automated Vision-Based Microsurgical Skill Analysis in Neurosurgery [12] 4. Classification of GB wall vascularity [68] 5. Language-based translation and prediction of surgical navigation steps for endoscopic wayfinding assistance in MIS [69] 6. A deep learning approach for pose estimation from volumetric OCT data [331] 7. Intra-abdominal acoustic feedback improves the perception during MIS [76] 8. Representation of instrument images in laparoscopy videos [81] 9. Application of Advanced Bioinformatics to Understand and Predict Burnout Among Surgical Trainees [107] 10. Multi-instance multi-label annotation for LC images [83] 11. "Deep-Onto" network for surgical workflow and context recognition [196] 12. Surgical SATR-DL and Task Recognition In RAS [95] 13. Combination of GAN and CNN for automatic subcentimeter pulmonary adenocarcinoma classification [332] 14. Context aware decision support in neurosurgical oncology based on an efficient classification of endomicroscopic data [98] Towards Retrieving Force Feedback in RAS: A Supervised Neuro-Recurrent-Vision Approach [258] |
| 34 | **Ridge regression** | 1. Evaluating Discrimination of ACS-NSQIP Surgical Risk Calculator in Thyroidectomy Patients [333] 2. Rib Fracture Frailty Index: A risk stratification tool for geriatric patients with multiple rib fractures [334] 3. Preoperative Prediction of Metastasis for Ovarian Cancer Based on Computed Tomography Radiomics Features and Clinical Factors [335] 4. Hospital-Based Back Surgery: Geospatial-Temporal, Explanatory, and Predictive Models [336] 5. A Machine Learning Approach to Predicting Case Duration for Robot-Assisted Surgery [243] 6. Prediction of soft tissue deformations after CMF surgery with incremental kernel ridge regression [337] |
| 35 | **Semantic segmentation** | 1. Automatic Segmentation of Oral and Oropharyngeal Cancer Using NBI [117] 2. Artificial Intelligence for Intraoperative Guidance: Using Semantic Segmentation to Identify Surgical Anatomy During LC [8] 3. Unravelling the effect of data augmentation transformations in polyp segmentation [338] 4. Multi-level feature aggregation network for instrument identification of endoscopic images [132] 5. Learned optical flow for intra-operative tracking of the retinal fundus [134] 6. Weakly supervised segmentation for real-time surgical tool tracking [28] 7. Deep learning for computerized laparoscopic image understanding in gynecology [138] 8. State recognition of decompressive laminectomy with multiple information in RAS [257] 9. A Modified Encoder-Decoder U-Net Architecture for Semantic and Instance Segmentation of Surgical Instruments from Laparoscopic Images [139] 10. A dataset of laryngeal endoscopic images with comparative study on convolution neural network-based semantic segmentation [155] 11. Keyframe extraction from laparoscopic videos based on visual saliency detection [265] |
| 36 | **Supervised learning** | 1. Unsupervised Monocular Depth Estimation for Colonoscope System Using Feedback Network [339] 2. Cross-modal learning for gesture and skill recognition in robotic surgery [62] 3. Deep Learning-Based Haptic Guidance for Surgical Skills Transfer [255] 4. Drivers of Cost Associated with Minimally Invasive Esophagectomy [13] 5. LTHRM Big Data Functional Analysis [66] 6. Temporal variability of surgical technical skill perception in real robotic surgery [340] 7. Risk assessment for intra-abdominal injury following blunt trauma in children: Derivation and validation of a machine learning model [294] 8. Learned optical flow for intra-operative tracking of the retinal fundus [134] 9. Weakly-supervised convolutional neural networks of renal tumor segmentation in abdominal CTA images [201] 10. Weakly supervised segmentation for real-time surgical tool tracking [28] 11. Application of Advanced Bioinformatics to Understand and Predict Burnout Among Surgical Trainees [107] 12. Towards near real-time assessment of surgical skills: A comparison of feature extraction techniques [56] 13. A novel automated lumen segmentation and classification algorithm for detection of irregular protrusion after stents deployment [296] 14. DRS of human joint tissue [85] 15. A DSS for large kidney stone treatment [88] 16. Novel evaluation of surgical activity recognition models using task-based efficiency metrics [147] 17. Weakly supervised convolutional LSTM approach for tool tracking in laparoscopic videos [151] 18. A dataset of laryngeal endoscopic images with comparative study on convolution neural network-based semantic segmentation [155] 19. A Machine Learning Approach to Predicting Case Duration for RAS [243] 20. Use of machine learning to predict early biochemical recurrence after robot-assisted prostatectomy [254] 21. Computer-assisted liver graft steatosis assessment via learning-based texture analysis [74] 22. Automatic selection of informative laryngoscopic-video frames [79] 23. Addressing multi-label imbalance problem of surgical tool detection using CNN [106] Towards Retrieving Force Feedback in RAS: A Supervised Neuro-Recurrent-Vision Approach [258] |
| 37 | **Support vector machines (SVM)** | 1. NLP surgical phase recognition in LC [63] 2. Automatic selection of informative laryngoscopic-video frames [78] 3. Energy-Based Metrics for Arthroscopic Skills Assessment [102] 4. Radiomics nomogram prediction of the malignant potential of GISTs [97] 5. Automated robot-assisted surgical skill evaluation: Predictive analytics approach [104] 6. Predictors of 30-Day Mortality Among Dutch Patients Undergoing CC, 2011-2016 [207] 7. Marginal radiomics features as imaging biomarkers for pathological invasion in lung adenocarcinoma [246] 8. Intraoperative margin assessment in oral and oropharyngeal cancer using FLIm [124] 9. Prediction of different outcome variables of PCNL [341] 10. A GC LncRNAs model for MSI and survival prediction based on support vector machine [342] 11. Predicting the quality of surgical exposure using spatial and procedural features from laparoscopic videos [108] 12. Vessel pattern characterization of larynx via CE + NBI images [86] 13. A DSS for large kidney stone treatment [88] 14. Resectability prediction of HIPEC [91] 15. Automatic and near real-time stylistic behavior assessment in robotic surgery [343] 16. Hyperspectral imaging for tissue classification, a way toward smart LCRS [94] 17. Discrimination between arterial and venous bowel ischemia by computer-assisted analysis of the fluorescent signal [344] 18. HMI for intuitive vision control and continuous surgical operation [96] 19. Learning-based classification of informative laryngoscopic frames [79] 20. Real-time tissue deformation in image-guided neurosurgery [51] 21. Objective Assessment of Endovascular Navigation Skills with Force Sensing [345] |
| 38 | **Transition state clustering (TSC)** | 1. Tumour budding, poorly differentiated clusters, and T-cell response in CC [346] 2. Elevated Colonic Mucin Expression Correlates with Extended Time to Surgery for Ulcerative Colitis Patients [347] 3. Restating Surgical Risk: From Patient to Population [348] |
| 39 | **Unsupervised learning** | 1. Unsupervised Monocular Depth Estimation for Colonoscope System Using Feedback Network [339] 2. Recovering dense 3D point clouds from single endoscopic image [54] 3. See-Through Vision with Unsupervised Scene Occlusion Reconstruction [121] 4. Application of Advanced Bioinformatics to Understand and Predict Burnout Among Surgical Trainees [107] 5. Generative Cooperative Networks for Joint Surgical Smoke Detection and Removal [143] 6. AR navigation for liver resection with a stereoscopic laparoscope [146] 7. Unsupervised binocular depth prediction network for LS [349] 8. Computer Aided Nodule Analysis and Risk Yield (CANARY) characterization of adenocarcinoma: radiologic biopsy, risk stratification and future directions [350] 9. Unsupervised Reverse Domain Adaptation for Synthetic Medical Images via Adversarial Training [206] |

**Abbreviations:**

AIRAM= Artificial intelligence based real-time analysis microperfusion, AR= Augmented reality, BESS= biportal endoscopic spine surgery, CAD= computer-assisted diagnosis, CC= colorectal cancer, CD= Cushing's disease, CE= contact endoscopy, CVS= critical view of safety, DBS= Deep Brain Stimulation, DRS= diffuse reflectance spectroscopy, DSS*=* decision support system, EUS= endoscopic ultrasound, FCNN= Fully convoluted neural network, FESS= functional endoscopic sinus surgery, FLIm= Fluorescence Lifetime Imaging, FNAC= Fine needle aspiration cytology, GB= gallbladder, GC= gastric cancer, GIST= Gastrointestinal stromal tumor, GS-SP= granular sub-stitch phases, HMI= head-mounted master interface, HIPEC= Hyperthermic Intraperitoneal Chemotherapy, HIS= hyperspectral imaging, IONM= Intraoperative neurophysiological monitoring, LC= laparoscopic cholecystectomy, LCRS= laparoscopic colorectal surgery, LS= laparoscopic surgery, LRTD= long-range temporal dependency, LSTM= long short-term memory, LTHRM= long term high-resolution manometry, LVSG= laparoscopic vertical sleeve gastrectomy, MIE= minimally invasive esophagectomy, MIPN= minimally invasive partial nephrectomy, MIS= Minimally Invasive Surgery, MLFA-Net= multilevel feature-aggregated deep convolutional neural network, MRI= Magnetic Resonance Imaging, NBI= narrow band imaging, NLBPM= natural language-based prediction method, NIRS= near infrared reflectance spectroscopy optical imaging, NLP= natural language processing, OAGB= one-anastomosis gastric bypass, OC= optical coherence tomography, PA= pulmonary adenocarcinomas, PAWSS= Patch-based adaptive weighting with segmentation and scale, pCLE= Probe-based confocal laser endomicroscopy, PCNL= percutaneous nephrolithotomy, PM= Peritoneal metastasis, POEM= peroral endoscopic myotomy, RARP= robot-assisted radical prostatectomy, RS= robotic surgery, SATR-DL= Surgical Skill Assessment And Task Recognition- deep learning, SLNS= sentence-level navigation steps, STN= Subthalamic Nucleus, SVM= support vector machine, TEF= tissue endogenous fluorescence, TMJOA= temporomandibular joint osteoarthritis, TSS= transsphenoidal surgery, TTTS= Twin-to-Twin Transfusion Syndrome, VUA= vesico-urethral anastomosis.

## **Appendix References**

1. Chen AB, Liang S, Nguyen JH, Liu Y, Hung AJ (2021) Machine learning analyses of automated performance metrics during granular sub-stitch phases predict surgeon experience. Surgery 169:1245–1249. https://doi.org/10.1016/j.surg.2020.09.020

2. Zherebtsov E, Zajnulina M, Kandurova K, Potapova E, Dremin V, Mamoshin A, Sokolovski S, Dunaev A, Rafailov EU (2020) Machine Learning Aided Photonic Diagnostic System for Minimally Invasive Optically Guided Surgery in the Hepatoduodenal Area. Diagnostics 10:873. https://doi.org/10.3390/diagnostics10110873

3. Besler E, Curtis Wang Y, C. Chan T, V. Sahakian A (2019) Real-time monitoring radiofrequency ablation using tree-based ensemble learning models. International Journal of Hyperthermia 36:427–436. https://doi.org/10.1080/02656736.2019.1587008

4. Kim D, Hwang W, Bae J, Park H, Kim KG (2021) Video Archiving and Communication System (VACS): A Progressive Approach, Design, Implementation, and Benefits for Surgical Videos. Healthc Inform Res 27:162–167. https://doi.org/10.4258/hir.2021.27.2.162

5. Lorenzovici N, Dulf E-H, Mocan T, Mocan L (2021) Artificial Intelligence in Colorectal Cancer Diagnosis Using Clinical Data: Non-Invasive Approach. Diagnostics 11:514. https://doi.org/10.3390/diagnostics11030514

6. Korndorffer JR, Hawn MT, Spain DA, Knowlton LM, Azagury DE, Nassar AK, Lau JN, Arnow KD, Trickey AW, Pugh CM (2020) Situating Artificial Intelligence in Surgery: A Focus on Disease Severity. Annals of Surgery 272:523–528. https://doi.org/10.1097/SLA.0000000000004207

7. Mascagni P, Alapatt D, Urade T, Vardazaryan A, Mutter D, Marescaux J, Costamagna G, Dallemagne B, Padoy N (2021) A Computer Vision Platform to Automatically Locate Critical Events in Surgical Videos: Documenting Safety in Laparoscopic Cholecystectomy. Annals of Surgery 274:e93–e95. https://doi.org/10.1097/SLA.0000000000004736

8. Madani A, Namazi B, Altieri MS, Hashimoto DA, Rivera AM, Pucher PH, Navarrete-Welton A, Sankaranarayanan G, Brunt LM, Okrainec A, Alseidi A (2020) Artificial Intelligence for Intraoperative Guidance: Using Semantic Segmentation to Identify Surgical Anatomy During Laparoscopic Cholecystectomy. Annals of Surgery Publish Ahead of Print: https://doi.org/10.1097/SLA.0000000000004594

9. Hashimoto DA, Rosman G, Witkowski ER, Stafford C, Navarette-Welton AJ, Rattner DW, Lillemoe KD, Rus DL, Meireles OR (2019) Computer Vision Analysis of Intraoperative Video: Automated Recognition of Operative Steps in Laparoscopic Sleeve Gastrectomy. Annals of Surgery 270:414–421. https://doi.org/10.1097/SLA.0000000000003460

10. Schwarz L, Aloia TA, Eng C, Chang GJ, Vauthey JN, Conrad C (2016) Transthoracic Port Placement Increases Safety of Total Laparoscopic Posterior Sectionectomy. Ann Surg Oncol 23:2167–2167. https://doi.org/10.1245/s10434-016-5126-2

11. Mascagni P, Vardazaryan A, Alapatt D, Urade T, Emre T, Fiorillo C, Pessaux P, Mutter D, Marescaux J, Costamagna G, Dallemagne B, Padoy N (2020) Artificial Intelligence for Surgical Safety: Automatic Assessment of the Critical View of Safety in Laparoscopic Cholecystectomy Using Deep Learning. Annals of Surgery Publish Ahead of Print: https://doi.org/10.1097/SLA.0000000000004351

12. Davids J, Makariou S-G, Ashrafian H, Darzi A, Marcus HJ, Giannarou S (2021) Automated Vision-Based Microsurgical Skill Analysis in Neurosurgery Using Deep Learning: Development and Preclinical Validation. World Neurosurgery 149:e669–e686. https://doi.org/10.1016/j.wneu.2021.01.117

13. Panda N, Shagabayeva L, Comrie CE, Phan N, Moonsamy P, Jeffrey Yang C-F, Fernandez FG, Morse CR (2021) Drivers of Cost Associated With Minimally Invasive Esophagectomy. The Annals of Thoracic Surgery S0003497521001223. https://doi.org/10.1016/j.athoracsur.2021.01.023

14. Anteby R, Horesh N, Soffer S, Zager Y, Barash Y, Amiel I, Rosin D, Gutman M, Klang E (2021) Deep learning visual analysis in laparoscopic surgery: a systematic review and diagnostic test accuracy meta-analysis. Surg Endosc 35:1521–1533. https://doi.org/10.1007/s00464-020-08168-1

15. Jung W-J, Kwak K-S, Lim S-C (2020) Vision-Based Suture Tensile Force Estimation in Robotic Surgery. Sensors 21:110. https://doi.org/10.3390/s21010110

16. Ward TM, Mascagni P, Ban Y, Rosman G, Padoy N, Meireles O, Hashimoto DA (2021) Computer vision in surgery. Surgery 169:1253–1256. https://doi.org/10.1016/j.surg.2020.10.039

17. Machrowska A, Szabelski J, Karpiński R, Krakowski P, Jonak J, Jonak K (2020) Use of Deep Learning Networks and Statistical Modeling to Predict Changes in Mechanical Parameters of Contaminated Bone Cements. Materials 13:5419. https://doi.org/10.3390/ma13235419

18. Marzullo A, Moccia S, Catellani M, Calimeri F, Momi ED (2021) Towards realistic laparoscopic image generation using image-domain translation. Computer Methods and Programs in Biomedicine 200:105834. https://doi.org/10.1016/j.cmpb.2020.105834

19. Jiang Y, Chen C, Zhang X, Chen C, Zhou Y, Ni G, Muh S, Lemos S (2020) Shoulder muscle activation pattern recognition based on sEMG and machine learning algorithms. Computer Methods and Programs in Biomedicine 197:105721. https://doi.org/10.1016/j.cmpb.2020.105721

20. Ding H, Xia W, Zhang L, Mao Q, Cao B, Zhao Y, Xu L, Jiang F, Dong G (2020) CT-Based Deep Learning Model for Invasiveness Classification and Micropapillary Pattern Prediction Within Lung Adenocarcinoma. Front Oncol 10:1186. https://doi.org/10.3389/fonc.2020.01186

21. Ward TM, Hashimoto DA, Ban Y, Rattner DW, Inoue H, Lillemoe KD, Rus DL, Rosman G, Meireles OR (2021) Automated operative phase identification in peroral endoscopic myotomy. Surg Endosc 35:4008–4015. https://doi.org/10.1007/s00464-020-07833-9

22. Kitaguchi D, Takeshita N, Matsuzaki H, Oda T, Watanabe M, Mori K, Kobayashi E, Ito M (2020) Automated laparoscopic colorectal surgery workflow recognition using artificial intelligence: Experimental research. International Journal of Surgery 79:88–94. https://doi.org/10.1016/j.ijsu.2020.05.015

23. Mikhaylov A, Pimashkin A, Pigareva Y, Gerasimova S, Gryaznov E, Shchanikov S, Zuev A, Talanov M, Lavrov I, Demin V, Erokhin V, Lobov S, Mukhina I, Kazantsev V, Wu H, Spagnolo B (2020) Neurohybrid Memristive CMOS-Integrated Systems for Biosensors and Neuroprosthetics. Front Neurosci 14:358. https://doi.org/10.3389/fnins.2020.00358

24. Kang AR, Lee J, Jung W, Lee M, Park SY, Woo J, Kim SH (2020) Development of a prediction model for hypotension after induction of anesthesia using machine learning. PLoS ONE 15:e0231172. https://doi.org/10.1371/journal.pone.0231172

25. Huang Z, Liu D, Chen X, Yu P, Wu J, Song B, Hu J, Wu B (2020) Retrospective imaging studies of gastric cancer: Study protocol clinical trial (SPIRIT Compliant). Medicine 99:e19157. https://doi.org/10.1097/MD.0000000000019157

26. Hopkins BS, Mazmudar A, Driscoll C, Svet M, Goergen J, Kelsten M, Shlobin NA, Kesavabhotla K, Smith ZA, Dahdaleh NS (2020) Using artificial intelligence (AI) to predict postoperative surgical site infection: A retrospective cohort of 4046 posterior spinal fusions. Clinical Neurology and Neurosurgery 192:105718. https://doi.org/10.1016/j.clineuro.2020.105718

27. Zhao Z, Cai T, Chang F, Cheng X (2019) Real‐time surgical instrument detection in robot‐assisted surgery using a convolutional neural network cascade. Healthcare Technology Letters 6:275–279. https://doi.org/10.1049/htl.2019.0064

28. Lee E, Plishker W, Liu X, Bhattacharyya SS, Shekhar R (2019) Weakly supervised segmentation for real‐time surgical tool tracking. Healthcare Technology Letters 6:231–236. https://doi.org/10.1049/htl.2019.0083

29. Won D, Lee H-J, Lee S-J, Park SH (2020) Spinal Stenosis Grading in Magnetic Resonance Imaging Using Deep Convolutional Neural Networks. Spine 45:804–812. https://doi.org/10.1097/BRS.0000000000003377

30. Torun Y, Öztürk A (2020) A New Breakthrough Detection Method for Bone Drilling in Robotic Orthopedic Surgery with Closed-Loop Control Approach. Ann Biomed Eng 48:1218–1229. https://doi.org/10.1007/s10439-019-02444-5

31. Casella A, Moccia S, Frontoni E, Paladini D, De Momi E, Mattos LS (2020) Inter-foetus Membrane Segmentation for TTTS Using Adversarial Networks. Ann Biomed Eng 48:848–859. https://doi.org/10.1007/s10439-019-02424-9

32. Zhou J, Wu L, Wan X, Shen L, Liu J, Zhang J, Jiang X, Wang Z, Yu S, Kang J, Li M, Hu S, Hu X, Gong D, Chen D, Yao L, Zhu Y, Yu H (2020) A novel artificial intelligence system for the assessment of bowel preparation (with video). Gastrointestinal Endoscopy 91:428-435.e2. https://doi.org/10.1016/j.gie.2019.11.026

33. Wise ES, Amateau SK, Ikramuddin S, Leslie DB (2020) Prediction of thirty-day morbidity and mortality after laparoscopic sleeve gastrectomy: data from an artificial neural network. Surg Endosc 34:3590–3596. https://doi.org/10.1007/s00464-019-07130-0

34. Mendizabal A, Sznitman R, Cotin S (2019) Force classification during robotic interventions through simulation-trained neural networks. Int J CARS 14:1601–1610. https://doi.org/10.1007/s11548-019-02048-3

35. Shoukri B, Prieto JC, Ruellas A, Yatabe M, Sugai J, Styner M, Zhu H, Huang C, Paniagua B, Aronovich S, Ashman L, Benavides E, de Dumast P, Ribera NT, Mirabel C, Michoud L, Allohaibi Z, Ioshida M, Bittencourt L, Fattori L, Gomes LR, Cevidanes L (2019) Minimally Invasive Approach for Diagnosing TMJ Osteoarthritis. J Dent Res 98:1103–1111. https://doi.org/10.1177/0022034519865187

36. Unberath M, Zaech J-N, Gao C, Bier B, Goldmann F, Lee SC, Fotouhi J, Taylor R, Armand M, Navab N (2019) Enabling machine learning in X-ray-based procedures via realistic simulation of image formation. Int J CARS 14:1517–1528. https://doi.org/10.1007/s11548-019-02011-2

37. Sheikhtaheri A, Orooji A, Pazouki A, Beitollahi M (2019) A Clinical Decision Support System for Predicting the Early Complications of One-Anastomosis Gastric Bypass Surgery. OBES SURG 29:2276–2286. https://doi.org/10.1007/s11695-019-03849-w

38. Hung AJ, Chen J, Ghodoussipour S, Oh PJ, Liu Z, Nguyen J, Purushotham S, Gill IS, Liu Y (2019) A deep-learning model using automated performance metrics and clinical features to predict urinary continence recovery after robot-assisted radical prostatectomy. BJU Int 124:487–495. https://doi.org/10.1111/bju.14735

39. Kowalewski K-F, Garrow CR, Schmidt MW, Benner L, Müller-Stich BP, Nickel F (2019) Sensor-based machine learning for workflow detection and as key to detect expert level in laparoscopic suturing and knot-tying. Surg Endosc 33:3732–3740. https://doi.org/10.1007/s00464-019-06667-4

40. Al‐Abdullah KI, Lim CP, Najdovski Z, Yassin W (2019) A model‐based bone milling state identification method via force sensing for a robotic surgical system. Int J Med Robotics Comput Assist Surg 15:. https://doi.org/10.1002/rcs.1989

41. Zhu Y, Wang Q-C, Xu M-D, Zhang Z, Cheng J, Zhong Y-S, Zhang Y-Q, Chen W-F, Yao L-Q, Zhou P-H, Li Q-L (2019) Application of convolutional neural network in the diagnosis of the invasion depth of gastric cancer based on conventional endoscopy. Gastrointestinal Endoscopy 89:806-815.e1. https://doi.org/10.1016/j.gie.2018.11.011

42. Laverde R, Rueda C, Amado L, Rojas D, Altuve M (2018) Artificial Neural Network for Laparoscopic Skills Classification Using Motion Signals from Apple Watch. In: 2018 40th Annual International Conference of the IEEE Engineering in Medicine and Biology Society (EMBC). IEEE, Honolulu, HI, pp 5434–5437

43. Stephens TK, O’Neill JJ, Kong NJ, Mazzeo MV, Norfleet JE, Sweet RM, Kowalewski TM (2019) Conditions for reliable grip force and jaw angle estimation of da Vinci surgical tools. Int J CARS 14:117–127. https://doi.org/10.1007/s11548-018-1866-8

44. Golinelli D, Bucci A, Toscano F, Filicori F, Fantini MP (2018) Real and predicted mortality under health spending constraints in Italy: a time trend analysis through artificial neural networks. BMC Health Serv Res 18:671. https://doi.org/10.1186/s12913-018-3473-3

45. Kastalskiy I, Mironov V, Lobov S, Krilova N, Pimashkin A, Kazantsev V (2018) A Neuromuscular Interface for Robotic Devices Control. Computational and Mathematical Methods in Medicine 2018:1–8. https://doi.org/10.1155/2018/8948145

46. Lin J, Clancy NT, Qi J, Hu Y, Tatla T, Stoyanov D, Maier-Hein L, Elson DS (2018) Dual-modality endoscopic probe for tissue surface shape reconstruction and hyperspectral imaging enabled by deep neural networks. Medical Image Analysis 48:162–176. https://doi.org/10.1016/j.media.2018.06.004

47. Narayan J, Singla E, Soni S, Singla A (2018) Adaptive neuro-fuzzy inference system–based path planning of 5-degrees-of-freedom spatial manipulator for medical applications. Proc Inst Mech Eng H 232:726–732. https://doi.org/10.1177/0954411918781418

48. Alonso-Silverio GA, Pérez-Escamirosa F, Bruno-Sanchez R, Ortiz-Simon JL, Muñoz-Guerrero R, Minor-Martinez A, Alarcón-Paredes A (2018) Development of a Laparoscopic Box Trainer Based on Open Source Hardware and Artificial Intelligence for Objective Assessment of Surgical Psychomotor Skills. Surg Innov 25:380–388. https://doi.org/10.1177/1553350618777045

49. Uemura M, Tomikawa M, Miao T, Souzaki R, Ieiri S, Akahoshi T, Lefor AK, Hashizume M (2018) Feasibility of an AI-Based Measure of the Hand Motions of Expert and Novice Surgeons. Computational and Mathematical Methods in Medicine 2018:1–6. https://doi.org/10.1155/2018/9873273

50. Sun Z, Maréchal L, Foong S (2018) Passive magnetic-based localization for precise untethered medical instrument tracking. Computer Methods and Programs in Biomedicine 156:151–161. https://doi.org/10.1016/j.cmpb.2017.12.018

51. Tonutti M, Gras G, Yang G-Z (2017) A machine learning approach for real-time modelling of tissue deformation in image-guided neurosurgery. Artificial Intelligence in Medicine 80:39–47. https://doi.org/10.1016/j.artmed.2017.07.004

52. Aminsharifi A, Irani D, Pooyesh S, Parvin H, Dehghani S, Yousofi K, Fazel E, Zibaie F (2017) Artificial Neural Network System to Predict the Postoperative Outcome of Percutaneous Nephrolithotomy. Journal of Endourology 31:461–467. https://doi.org/10.1089/end.2016.0791

53. Ekkachai K, Nilkhamhang I (2016) Swing Phase Control of Semi-Active Prosthetic Knee Using Neural Network Predictive Control With Particle Swarm Optimization. IEEE Trans Neural Syst Rehabil Eng 24:1169–1178. https://doi.org/10.1109/TNSRE.2016.2521686

54. Xi L, Zhao Y, Chen L, Gao QH, Tang W, Wan TR, Xue T (2021) Recovering dense 3D point clouds from single endoscopic image. Computer Methods and Programs in Biomedicine 205:106077. https://doi.org/10.1016/j.cmpb.2021.106077

55. Shehata M, Shalaby A, Switala AE, El‐Baz M, Ghazal M, Fraiwan L, Khalil A, El‐Ghar MA, Badawy M, Bakr AM, Dwyer A, Elmaghraby A, Giridharan G, Keynton R, El‐Baz A (2020) A multimodal computer‐aided diagnostic system for precise identification of renal allograft rejection: Preliminary results. Med Phys 47:2427–2440. https://doi.org/10.1002/mp.14109

56. Anh NX, Nataraja RM, Chauhan S (2020) Towards near real-time assessment of surgical skills: A comparison of feature extraction techniques. Computer Methods and Programs in Biomedicine 187:105234. https://doi.org/10.1016/j.cmpb.2019.105234

57. Cerveri P, Belfatto A, Baroni G, Manzotti A (2018) Stacked sparse autoencoder networks and statistical shape models for automatic staging of distal femur trochlear dysplasia. Int J Med Robotics Comput Assist Surg 14:e1947. https://doi.org/10.1002/rcs.1947

58. Ramesh S, Dall’Alba D, Gonzalez C, Yu T, Mascagni P, Mutter D, Marescaux J, Fiorini P, Padoy N (2021) Multi-task temporal convolutional networks for joint recognition of surgical phases and steps in gastric bypass procedures. Int J CARS 16:1111–1119. https://doi.org/10.1007/s11548-021-02388-z

59. Zha X, Wehbe L, Sclabassi RJ, Mace Z, Liang YV, Yu A, Leonardo J, Cheng BC, Hillman TA, Chen DA, Riviere CN (2021) A Deep Learning Model for Automated Classification of Intraoperative Continuous EMG. IEEE Trans Med Robot Bionics 3:44–52. https://doi.org/10.1109/TMRB.2020.3048255

60. Bombieri M, Rospocher M, Dall’Alba D, Fiorini P (2021) Automatic detection of procedural knowledge in robotic-assisted surgical texts. Int J CARS 16:1287–1295. https://doi.org/10.1007/s11548-021-02370-9

61. Kummert J, Schulz A, Redick T, Ayoub N, Modabber A, Abel D, Hammer B (2021) Efficient Reject Options for Particle Filter Object Tracking in Medical Applications. Sensors 21:2114. https://doi.org/10.3390/s21062114

62. Wu JY, Tamhane A, Kazanzides P, Unberath M (2021) Cross-modal self-supervised representation learning for gesture and skill recognition in robotic surgery. Int J CARS 16:779–787. https://doi.org/10.1007/s11548-021-02343-y

63. Guzmán-García C, Gómez-Tome M, Sánchez-González P, Oropesa I, Gómez EJ (2021) Speech-Based Surgical Phase Recognition for Non-Intrusive Surgical Skills’ Assessment in Educational Contexts. Sensors 21:1330. https://doi.org/10.3390/s21041330

64. Aikemu B, Xue P, Hong H, Jia H, Wang C, Li S, Huang L, Ding X, Zhang H, Cai G, Lu A, Xie L, Li H, Zheng M, Sun J (2021) Artificial Intelligence in Decision-Making for Colorectal Cancer Treatment Strategy: An Observational Study of Implementing Watson for Oncology in a 250-Case Cohort. Front Oncol 10:594182. https://doi.org/10.3389/fonc.2020.594182

65. Keles HO, Cengiz C, Demiral I, Ozmen MM, Omurtag A (2021) High density optical neuroimaging predicts surgeons’s subjective experience and skill levels. PLoS ONE 16:e0247117. https://doi.org/10.1371/journal.pone.0247117

66. Jell A, Kuttler C, Ostler D, Hüser N (2020) How to Cope with Big Data in Functional Analysis of the Esophagus. Visc Med 36:439–442. https://doi.org/10.1159/000511931

67. Park S-H, Park H-M, Baek K-R, Ahn H-M, Lee IY, Son GM (2020) Artificial intelligence based real-time microcirculation analysis system for laparoscopic colorectal surgery. WJG 26:6945–6962. https://doi.org/10.3748/wjg.v26.i44.6945

68. Loukas C, Frountzas M, Schizas D (2021) Patch-based classification of gallbladder wall vascularity from laparoscopic images using deep learning. Int J CARS 16:103–113. https://doi.org/10.1007/s11548-020-02285-x

69. Bieck R, Heuermann K, Pirlich M, Neumann J, Neumuth T (2020) Language-based translation and prediction of surgical navigation steps for endoscopic wayfinding assistance in minimally invasive surgery. Int J CARS 15:2089–2100. https://doi.org/10.1007/s11548-020-02264-2

70. Kelly JD, Petersen A, Lendvay TS, Kowalewski TM (2020) Bidirectional long short-term memory for surgical skill classification of temporally segmented tasks. Int J CARS 15:2079–2088. https://doi.org/10.1007/s11548-020-02269-x

71. Sharma C, Singh H, Orihuela-Espina F, Darzi A, Sodergren MH (2021) Visual gaze patterns reveal surgeons’ ability to identify risk of bile duct injury during laparoscopic cholecystectomy. HPB 23:715–722. https://doi.org/10.1016/j.hpb.2020.09.007

72. Luongo F, Hakim R, Nguyen JH, Anandkumar A, Hung AJ (2021) Deep learning-based computer vision to recognize and classify suturing gestures in robot-assisted surgery. Surgery 169:1240–1244. https://doi.org/10.1016/j.surg.2020.08.016

73. Cesaretti M, Brustia R, Goumard C, Cauchy F, Poté N, Dondero F, Paugam‐Burtz C, Durand F, Paradis V, Diaspro A, Mattos L, Scatton O, Soubrane O, Moccia S (2020) Use of Artificial Intelligence as an Innovative Method for Liver Graft Macrosteatosis Assessment. Liver Transpl 26:1224–1232. https://doi.org/10.1002/lt.25801

74. Moccia S, Mattos LS, Patrini I, Ruperti M, Poté N, Dondero F, Cauchy F, Sepulveda A, Soubrane O, De Momi E, Diaspro A, Cesaretti M (2018) Computer-assisted liver graft steatosis assessment via learning-based texture analysis. Int J CARS 13:1357–1367. https://doi.org/10.1007/s11548-018-1787-6

75. Zhang J, Zhu L, Yao L, Ding X, Chen D, Wu H, Lu Z, Zhou W, Zhang L, An P, Xu B, Tan W, Hu S, Cheng F, Yu H (2020) Deep learning–based pancreas segmentation and station recognition system in EUS: development and validation of a useful training tool (with video). Gastrointestinal Endoscopy 92:874-885.e3. https://doi.org/10.1016/j.gie.2020.04.071

76. Ostler D, Seibold M, Fuchtmann J, Samm N, Feussner H, Wilhelm D, Navab N (2020) Acoustic signal analysis of instrument–tissue interaction for minimally invasive interventions. Int J CARS 15:771–779. https://doi.org/10.1007/s11548-020-02146-7

77. Khosravi M, Atashzar SF, Gilmore G, Jog MS, Patel RV (2020) Intraoperative Localization of STN During DBS Surgery Using a Data-Driven Model. IEEE J Transl Eng Health Med 8:1–9. https://doi.org/10.1109/JTEHM.2020.2969152

78. Patrini I, Ruperti M, Moccia S, Mattos LS, Frontoni E, De Momi E (2020) Transfer learning for informative-frame selection in laryngoscopic videos through learned features. Med Biol Eng Comput 58:1225–1238. https://doi.org/10.1007/s11517-020-02127-7

79. Moccia S, Vanone GO, Momi ED, Laborai A, Guastini L, Peretti G, Mattos LS (2018) Learning-based classification of informative laryngoscopic frames. Computer Methods and Programs in Biomedicine 158:21–30. https://doi.org/10.1016/j.cmpb.2018.01.030

80. Barczyński M, Stopa-Barczyńska M, Wojtczak B, Czarniecka A, Konturek A (2020) Clinical validation of S-DetectTM mode in semi-automated ultrasound classification of thyroid lesions in surgical office. Gland Surg 9:S77–S85. https://doi.org/10.21037/gs.2019.12.23

81. Kletz S, Schoeffmann K, Husslein H (2019) Learning the representation of instrument images in laparoscopy videos. Healthcare Technology Letters 6:197–203. https://doi.org/10.1049/htl.2019.0077

82. Kott O, Linsley D, Amin A, Karagounis A, Jeffers C, Golijanin D, Serre T, Gershman B (2021) Development of a Deep Learning Algorithm for the Histopathologic Diagnosis and Gleason Grading of Prostate Cancer Biopsies: A Pilot Study. European Urology Focus 7:347–351. https://doi.org/10.1016/j.euf.2019.11.003

83. Loukas C, Sgouros NP (2020) Multi‐instance multi‐label learning for surgical image annotation. Int J Med Robot 16:. https://doi.org/10.1002/rcs.2058

84. Wu C, Cha J, Sulek J, Zhou T, Sundaram CP, Wachs J, Yu D (2020) Eye-Tracking Metrics Predict Perceived Workload in Robotic Surgical Skills Training. Hum Factors 62:1365–1386. https://doi.org/10.1177/0018720819874544

85. Gunaratne R, Monteath I, Goncalves J, Sheh R, Ironside CN, Kapfer M, Chipper R, Robertson B, Khan R, Fick D (2019) Machine learning classification of human joint tissue from diffuse reflectance spectroscopy data. Biomed Opt Express 10:3889. https://doi.org/10.1364/BOE.10.003889

86. Esmaeili N, Illanes A, Boese A, Davaris N, Arens C, Friebe M (2019) Novel automated vessel pattern characterization of larynx contact endoscopic video images. Int J CARS 14:1751–1761. https://doi.org/10.1007/s11548-019-02034-9

87. Kannan S, Yengera G, Mutter D, Marescaux J, Padoy N (2020) Future-State Predicting LSTM for Early Surgery Type Recognition. IEEE Trans Med Imaging 39:556–566. https://doi.org/10.1109/TMI.2019.2931158

88. Shabaniyan T, Parsaei H, Aminsharifi A, Movahedi MM, Jahromi AT, Pouyesh S, Parvin H (2019) An artificial intelligence-based clinical decision support system for large kidney stone treatment. Australas Phys Eng Sci Med 42:771–779. https://doi.org/10.1007/s13246-019-00780-3

89. Nguyen XA, Ljuhar D, Pacilli M, Nataraja RM, Chauhan S (2019) Surgical skill levels: Classification and analysis using deep neural network model and motion signals. Computer Methods and Programs in Biomedicine 177:1–8. https://doi.org/10.1016/j.cmpb.2019.05.008

90. Du X, Allan M, Bodenstedt S, Maier-Hein L, Speidel S, Dore A, Stoyanov D (2019) Patch-based adaptive weighting with segmentation and scale (PAWSS) for visual tracking in surgical video. Medical Image Analysis 57:120–135. https://doi.org/10.1016/j.media.2019.07.002

91. Maubert A, Birtwisle L, Bernard JL, Benizri E, Bereder JM (2019) Can machine learning predict resecability of a peritoneal carcinomatosis? Surgical Oncology 29:120–125. https://doi.org/10.1016/j.suronc.2019.04.008

92. Funke I, Mees ST, Weitz J, Speidel S (2019) Video-based surgical skill assessment using 3D convolutional neural networks. Int J CARS 14:1217–1225. https://doi.org/10.1007/s11548-019-01995-1

93. Saikia AR, Bora K, Mahanta LB, Das AK (2019) Comparative assessment of CNN architectures for classification of breast FNAC images. Tissue and Cell 57:8–14. https://doi.org/10.1016/j.tice.2019.02.001

94. Baltussen EJM, Kok END, Brouwer de Koning SG, Sanders J, Aalbers AGJ, Kok NFM, Beets GL, Flohil CC, Bruin SC, Kuhlmann KFD, Sterenborg HJCM, Ruers TJM (2019) Hyperspectral imaging for tissue classification, a way toward smart laparoscopic colorectal surgery. J Biomed Opt 24:1. https://doi.org/10.1117/1.JBO.24.1.016002

95. Wang Z, Fey AM (2018) SATR-DL: Improving Surgical Skill Assessment And Task Recognition In Robot-Assisted Surgery With Deep Neural Networks. In: 2018 40th Annual International Conference of the IEEE Engineering in Medicine and Biology Society (EMBC). IEEE, Honolulu, HI, pp 1793–1796

96. Hong N, Kim M, Lee C, Kim S (2019) Head-mounted interface for intuitive vision control and continuous surgical operation in a surgical robot system. Med Biol Eng Comput 57:601–614. https://doi.org/10.1007/s11517-018-1902-4

97. Chen T, Ning Z, Xu L, Feng X, Han S, Roth HR, Xiong W, Zhao X, Hu Y, Liu H, Yu J, Zhang Y, Li Y, Xu Y, Mori K, Li G (2019) Radiomics nomogram for predicting the malignant potential of gastrointestinal stromal tumours preoperatively. Eur Radiol 29:1074–1082. https://doi.org/10.1007/s00330-018-5629-2

98. Li Y, Charalampaki P, Liu Y, Yang G-Z, Giannarou S (2018) Context aware decision support in neurosurgical oncology based on an efficient classification of endomicroscopic data. Int J CARS 13:1187–1199. https://doi.org/10.1007/s11548-018-1806-7

99. Shafiei SB, Hussein AA, Muldoon SF, Guru KA (2018) Functional Brain States Measure Mentor-Trainee Trust during Robot-Assisted Surgery. Sci Rep 8:3667. https://doi.org/10.1038/s41598-018-22025-1

100. Ahmad J, Muhammad K, Lee MY, Baik SW (2017) Endoscopic Image Classification and Retrieval using Clustered Convolutional Features. J Med Syst 41:196. https://doi.org/10.1007/s10916-017-0836-y

101. Sgouros NP, Loukas C, Koufi V, Troupis TG, Georgiou E (2018) An automated skills assessment framework for laparoscopic training tasks. Int J Med Robotics Comput Assist Surg 14:e1853. https://doi.org/10.1002/rcs.1853

102. Poursartip B, LeBel M-E, McCracken L, Escoto A, Patel R, Naish M, Trejos A (2017) Energy-Based Metrics for Arthroscopic Skills Assessment. Sensors 17:1808. https://doi.org/10.3390/s17081808

103. Garcia-Martinez A, Vicente-Samper JM, Sabater-Navarro JM (2017) Automatic detection of surgical haemorrhage using computer vision. Artificial Intelligence in Medicine 78:55–60. https://doi.org/10.1016/j.artmed.2017.06.002

104. Fard MJ, Ameri S, Darin Ellis R, Chinnam RB, Pandya AK, Klein MD (2018) Automated robot-assisted surgical skill evaluation: Predictive analytics approach. Int J Med Robotics Comput Assist Surg 14:e1850. https://doi.org/10.1002/rcs.1850

105. French A, Lendvay TS, Sweet RM, Kowalewski TM (2017) Predicting surgical skill from the first N seconds of a task: value over task time using the isogony principle. Int J CARS 12:1161–1170. https://doi.org/10.1007/s11548-017-1606-5

106. Sahu M, Mukhopadhyay A, Szengel A, Zachow S (2017) Addressing multi-label imbalance problem of surgical tool detection using CNN. Int J CARS 12:1013–1020. https://doi.org/10.1007/s11548-017-1565-x

107. Kurbatov V, Shaughnessy M, Baratta V, Heller DR, Freedman-Weiss M, Resio BJ, Fleming M, Yoo PS (2020) Application of Advanced Bioinformatics to Understand and Predict Burnout Among Surgical Trainees. Journal of Surgical Education 77:499–507. https://doi.org/10.1016/j.jsurg.2019.11.008

108. Derathé A, Reche F, Moreau-Gaudry A, Jannin P, Gibaud B, Voros S (2020) Predicting the quality of surgical exposure using spatial and procedural features from laparoscopic videos. Int J CARS 15:59–67. https://doi.org/10.1007/s11548-019-02072-3

109. Feng Y, McGowan H, Semsar A, Zahiri HR, George IM, Park A, Kleinsmith A, Mentis H (2020) Virtual pointer for gaze guidance in laparoscopic surgery. Surg Endosc 34:3533–3539. https://doi.org/10.1007/s00464-019-07141-x

110. Ismail Fawaz H, Forestier G, Weber J, Idoumghar L, Muller P-A (2019) Accurate and interpretable evaluation of surgical skills from kinematic data using fully convolutional neural networks. Int J CARS 14:1611–1617. https://doi.org/10.1007/s11548-019-02039-4

111. Stroop R, Nakamura M, Schoukens J, Oliva Uribe D (2019) Tactile sensor-based real-time clustering for tissue differentiation. Int J CARS 14:129–137. https://doi.org/10.1007/s11548-018-1869-5

112. Omisore OM, Han S, Ren L, Elazab A, Hui L, Abdelhamid T, Azeez NA, Wang L (2018) Deeply-learnt damped least-squares (DL-DLS) method for inverse kinematics of snake-like robots. Neural Networks 107:34–47. https://doi.org/10.1016/j.neunet.2018.06.018

113. Zhang M, Cheng X, Copeland D, Desai A, Guan MY, Brat GA, Yeung S (2020) Using Computer Vision to Automate Hand Detection and Tracking of Surgeon Movements in Videos of Open Surgery. AMIA Annu Symp Proc 2020:1373–1382

114. Podlasek J, Heesch M, Podlasek R, Kilisiński W, Filip R (2021) Real-time deep learning-based colorectal polyp localization on clinical video footage achievable with a wide array of hardware configurations. Endosc Int Open 09:E741–E748. https://doi.org/10.1055/a-1388-6735

115. Michelin AM, Korres G, Ba’ara S, Assadi H, Alsuradi H, Sayegh RR, Argyros A, Eid M (2021) FaceGuard: A Wearable System To Avoid Face Touching. Front Robot AI 8:612392. https://doi.org/10.3389/frobt.2021.612392

116. Cho SM, Kim Y-G, Jeong J, Kim I, Lee H, Kim N (2021) Automatic tip detection of surgical instruments in biportal endoscopic spine surgery. Computers in Biology and Medicine 133:104384. https://doi.org/10.1016/j.compbiomed.2021.104384

117. Paderno A, Piazza C, Del Bon F, Lancini D, Tanagli S, Deganello A, Peretti G, De Momi E, Patrini I, Ruperti M, Mattos LS, Moccia S (2021) Deep Learning for Automatic Segmentation of Oral and Oropharyngeal Cancer Using Narrow Band Imaging: Preliminary Experience in a Clinical Perspective. Front Oncol 11:626602. https://doi.org/10.3389/fonc.2021.626602

118. Cui P, Shu T, Lei J, Chen W (2021) Nerve recognition in percutaneous transforaminal endoscopic discectomy using convolutional neural network. Med Phys 48:2279–2288. https://doi.org/10.1002/mp.14822

119. Lavanchy JL, Zindel J, Kirtac K, Twick I, Hosgor E, Candinas D, Beldi G (2021) Automation of surgical skill assessment using a three-stage machine learning algorithm. Sci Rep 11:5197. https://doi.org/10.1038/s41598-021-84295-6

120. Hasan MdK, Calvet L, Rabbani N, Bartoli A (2021) Detection, segmentation, and 3D pose estimation of surgical tools using convolutional neural networks and algebraic geometry. Medical Image Analysis 70:101994. https://doi.org/10.1016/j.media.2021.101994

121. Tukra S, Marcus HJ, Giannarou S (2021) See-Through Vision with Unsupervised Scene Occlusion Reconstruction. IEEE Trans Pattern Anal Mach Intell 1–1. https://doi.org/10.1109/TPAMI.2021.3058410

122. Namazi B, Sankaranarayanan G, Devarajan V (2021) A contextual detector of surgical tools in laparoscopic videos using deep learning. Surg Endosc. https://doi.org/10.1007/s00464-021-08336-x

123. Huang Z, Liu D, Chen X, He D, Yu P, Liu B, Wu B, Hu J, Song B (2020) Deep Convolutional Neural Network Based on Computed Tomography Images for the Preoperative Diagnosis of Occult Peritoneal Metastasis in Advanced Gastric Cancer. Front Oncol 10:601869. https://doi.org/10.3389/fonc.2020.601869

124. Marsden M, Weyers BW, Bec J, Sun T, Gandour-Edwards RF, Birkeland AC, Abouyared M, Bewley AF, Farwell DG, Marcu L (2021) Intraoperative Margin Assessment in Oral and Oropharyngeal Cancer Using Label-Free Fluorescence Lifetime Imaging and Machine Learning. IEEE Trans Biomed Eng 68:857–868. https://doi.org/10.1109/TBME.2020.3010480

125. Ling T, Wu L, Fu Y, Xu Q, An P, Zhang J, Hu S, Chen Y, He X, Wang J, Chen X, Zhou J, Xu Y, Zou X, Yu H (2021) A deep learning-based system for identifying differentiation status and delineating the margins of early gastric cancer in magnifying narrow-band imaging endoscopy. Endoscopy 53:469–477. https://doi.org/10.1055/a-1229-0920

126. Yu L, Wang P, Yan Y, Xia Y, Cao W (2020) MASSD: Multi-scale attention single shot detector for surgical instruments. Computers in Biology and Medicine 123:103867. https://doi.org/10.1016/j.compbiomed.2020.103867

127. Chen M, Wang J, Xiao Y, Wu L, Hu S, Chen S, Yi G, Hu W, Xie X, Zhu Y, Chen Y, Yang Y, Yu H (2021) Automated and real-time validation of gastroesophageal varices under esophagogastroduodenoscopy using a deep convolutional neural network: a multicenter retrospective study (with video). Gastrointestinal Endoscopy 93:422-432.e3. https://doi.org/10.1016/j.gie.2020.06.058

128. Shi X, Jin Y, Dou Q, Heng P-A (2020) LRTD: long-range temporal dependency based active learning for surgical workflow recognition. Int J CARS 15:1573–1584. https://doi.org/10.1007/s11548-020-02198-9

129. François T, Calvet L, Madad Zadeh S, Saboul D, Gasparini S, Samarakoon P, Bourdel N, Bartoli A (2020) Detecting the occluding contours of the uterus to automatise augmented laparoscopy: score, loss, dataset, evaluation and user study. Int J CARS 15:1177–1186. https://doi.org/10.1007/s11548-020-02151-w

130. Ahmad MA, Ourak M, Gruijthuijsen C, Deprest J, Vercauteren T, Vander Poorten E (2020) Deep learning-based monocular placental pose estimation: towards collaborative robotics in fetoscopy. Int J CARS 15:1561–1571. https://doi.org/10.1007/s11548-020-02166-3

131. Bano S, Vasconcelos F, Vander Poorten E, Vercauteren T, Ourselin S, Deprest J, Stoyanov D (2020) FetNet: a recurrent convolutional network for occlusion identification in fetoscopic videos. Int J CARS 15:791–801. https://doi.org/10.1007/s11548-020-02169-0

132. Chu Y, Yang X, Li H, Ai D, Ding Y, Fan J, Song H, Yang J (2020) Multi-level feature aggregation network for instrument identification of endoscopic images. Phys Med Biol 65:165004. https://doi.org/10.1088/1361-6560/ab8dda

133. Cai T, Zhao Z (2020) Convolutional neural network-based surgical instrument detection. THC 28:81–88. https://doi.org/10.3233/THC-209009

134. Ravasio CS, Pissas T, Bloch E, Flores B, Jalali S, Stoyanov D, Cardoso JM, Da Cruz L, Bergeles C (2020) Learned optical flow for intra-operative tracking of the retinal fundus. Int J CARS 15:827–836. https://doi.org/10.1007/s11548-020-02160-9

135. Yamazaki Y, Kanaji S, Matsuda T, Oshikiri T, Nakamura T, Suzuki S, Hiasa Y, Otake Y, Sato Y, Kakeji Y (2020) Automated Surgical Instrument Detection from Laparoscopic Gastrectomy Video Images Using an Open Source Convolutional Neural Network Platform. Journal of the American College of Surgeons 230:725-732.e1. https://doi.org/10.1016/j.jamcollsurg.2020.01.037

136. Ma Q, Kobayashi E, Fan B, Nakagawa K, Sakuma I, Masamune K, Suenaga H (2020) Automatic 3D landmarking model using patch‐based deep neural networks for CT image of oral and maxillofacial surgery. Int J Med Robot 16:. https://doi.org/10.1002/rcs.2093

137. de la Fuente López E, Muñoz García Á, Santos del Blanco L, Fraile Marinero JC, Pérez Turiel J (2020) Automatic gauze tracking in laparoscopic surgery using image texture analysis. Computer Methods and Programs in Biomedicine 190:105378. https://doi.org/10.1016/j.cmpb.2020.105378

138. Madad Zadeh S, Francois T, Calvet L, Chauvet P, Canis M, Bartoli A, Bourdel N (2020) SurgAI: deep learning for computerized laparoscopic image understanding in gynaecology. Surg Endosc 34:5377–5383. https://doi.org/10.1007/s00464-019-07330-8

139. Kamrul Hasan SM, Linte CA (2019) U-NetPlus: A Modified Encoder-Decoder U-Net Architecture for Semantic and Instance Segmentation of Surgical Instruments from Laparoscopic Images. In: 2019 41st Annual International Conference of the IEEE Engineering in Medicine and Biology Society (EMBC). IEEE, Berlin, Germany, pp 7205–7211

140. Dunnhofer M, Antico M, Sasazawa F, Takeda Y, Camps S, Martinel N, Micheloni C, Carneiro G, Fontanarosa D (2020) Siam-U-Net: encoder-decoder siamese network for knee cartilage tracking in ultrasound images. Medical Image Analysis 60:101631. https://doi.org/10.1016/j.media.2019.101631

141. Mikada T, Kanno T, Kawase T, Miyazaki T, Kawashima K (2020) Three‐dimensional posture estimation of robot forceps using endoscope with convolutional neural network. Int J Med Robot 16:. https://doi.org/10.1002/rcs.2062

142. Kitaguchi D, Takeshita N, Matsuzaki H, Takano H, Owada Y, Enomoto T, Oda T, Miura H, Yamanashi T, Watanabe M, Sato D, Sugomori Y, Hara S, Ito M (2020) Real-time automatic surgical phase recognition in laparoscopic sigmoidectomy using the convolutional neural network-based deep learning approach. Surg Endosc 34:4924–4931. https://doi.org/10.1007/s00464-019-07281-0

143. Chen L, Tang W, John NW, Wan TR, Zhang JJ (2020) De-smokeGCN: Generative Cooperative Networks for Joint Surgical Smoke Detection and Removal. IEEE Trans Med Imaging 39:1615–1625. https://doi.org/10.1109/TMI.2019.2953717

144. Zhang Y, Xie D (2019) Detection and segmentation of multi-class artifacts in endoscopy. J Zhejiang Univ Sci B 20:1014–1020. https://doi.org/10.1631/jzus.B1900340

145. Jin Y, Li H, Dou Q, Chen H, Qin J, Fu C-W, Heng P-A (2020) Multi-task recurrent convolutional network with correlation loss for surgical video analysis. Medical Image Analysis 59:101572. https://doi.org/10.1016/j.media.2019.101572

146. Luo H, Yin D, Zhang S, Xiao D, He B, Meng F, Zhang Y, Cai W, He S, Zhang W, Hu Q, Guo H, Liang S, Zhou S, Liu S, Sun L, Guo X, Fang C, Liu L, Jia F (2020) Augmented reality navigation for liver resection with a stereoscopic laparoscope. Computer Methods and Programs in Biomedicine 187:105099. https://doi.org/10.1016/j.cmpb.2019.105099

147. Zia A, Guo L, Zhou L, Essa I, Jarc A (2019) Novel evaluation of surgical activity recognition models using task-based efficiency metrics. Int J CARS 14:2155–2163. https://doi.org/10.1007/s11548-019-02025-w

148. Zhao Y, Guo S, Wang Y, Cui J, Ma Y, Zeng Y, Liu X, Jiang Y, Li Y, Shi L, Xiao N (2019) A CNN-based prototype method of unstructured surgical state perception and navigation for an endovascular surgery robot. Med Biol Eng Comput 57:1875–1887. https://doi.org/10.1007/s11517-019-02002-0

149. Fuentes-Hurtado F, Kadkhodamohammadi A, Flouty E, Barbarisi S, Luengo I, Stoyanov D (2019) EasyLabels: weak labels for scene segmentation in laparoscopic videos. Int J CARS 14:1247–1257. https://doi.org/10.1007/s11548-019-02003-2

150. Bier B, Goldmann F, Zaech J-N, Fotouhi J, Hegeman R, Grupp R, Armand M, Osgood G, Navab N, Maier A, Unberath M (2019) Learning to detect anatomical landmarks of the pelvis in X-rays from arbitrary views. Int J CARS 14:1463–1473. https://doi.org/10.1007/s11548-019-01975-5

151. Nwoye CI, Mutter D, Marescaux J, Padoy N (2019) Weakly supervised convolutional LSTM approach for tool tracking in laparoscopic videos. Int J CARS 14:1059–1067. https://doi.org/10.1007/s11548-019-01958-6

152. Bodenstedt S, Wagner M, Mündermann L, Kenngott H, Müller-Stich B, Breucha M, Mees ST, Weitz J, Speidel S (2019) Prediction of laparoscopic procedure duration using unlabeled, multimodal sensor data. Int J CARS 14:1089–1095. https://doi.org/10.1007/s11548-019-01966-6

153. Wu L, Zhou W, Wan X, Zhang J, Shen L, Hu S, Ding Q, Mu G, Yin A, Huang X, Liu J, Jiang X, Wang Z, Deng Y, Liu M, Lin R, Ling T, Li P, Wu Q, Jin P, Chen J, Yu H (2019) A deep neural network improves endoscopic detection of early gastric cancer without blind spots. Endoscopy 51:522–531. https://doi.org/10.1055/a-0855-3532

154. Zhao Z, Chen Z, Voros S, Cheng X (2019) Real-time tracking of surgical instruments based on spatio-temporal context and deep learning. Computer Assisted Surgery 24:20–29. https://doi.org/10.1080/24699322.2018.1560097

155. Laves M-H, Bicker J, Kahrs LA, Ortmaier T (2019) A dataset of laryngeal endoscopic images with comparative study on convolution neural network-based semantic segmentation. Int J CARS 14:483–492. https://doi.org/10.1007/s11548-018-01910-0

156. Wang Z, Majewicz Fey A (2018) Deep learning with convolutional neural network for objective skill evaluation in robot-assisted surgery. Int J CARS 13:1959–1970. https://doi.org/10.1007/s11548-018-1860-1

157. Anas EMA, Mousavi P, Abolmaesumi P (2018) A deep learning approach for real time prostate segmentation in freehand ultrasound guided biopsy. Medical Image Analysis 48:107–116. https://doi.org/10.1016/j.media.2018.05.010

158. Du X, Kurmann T, Chang P-L, Allan M, Ourselin S, Sznitman R, Kelly JD, Stoyanov D (2018) Articulated Multi-Instrument 2-D Pose Estimation Using Fully Convolutional Networks. IEEE Trans Med Imaging 37:1276–1287. https://doi.org/10.1109/TMI.2017.2787672

159. Gessert N, Schlüter M, Schlaefer A (2018) A deep learning approach for pose estimation from volumetric OCT data. Medical Image Analysis 46:162–179. https://doi.org/10.1016/j.media.2018.03.002

160. Choi B, Jo K, Choi S, Choi J (2017) Surgical-tools detection based on Convolutional Neural Network in laparoscopic robot-assisted surgery. In: 2017 39th Annual International Conference of the IEEE Engineering in Medicine and Biology Society (EMBC). IEEE, Seogwipo, pp 1756–1759

161. Zhao Z, Voros S, Weng Y, Chang F, Li R (2017) Tracking-by-detection of surgical instruments in minimally invasive surgery via the convolutional neural network deep learning-based method. Computer Assisted Surgery 22:26–35. https://doi.org/10.1080/24699322.2017.1378777

162. Sarikaya D, Corso JJ, Guru KA (2017) Detection and Localization of Robotic Tools in Robot-Assisted Surgery Videos Using Deep Neural Networks for Region Proposal and Detection. IEEE Trans Med Imaging 36:1542–1549. https://doi.org/10.1109/TMI.2017.2665671

163. Twinanda AP, Shehata S, Mutter D, Marescaux J, de Mathelin M, Padoy N (2017) EndoNet: A Deep Architecture for Recognition Tasks on Laparoscopic Videos. IEEE Trans Med Imaging 36:86–97. https://doi.org/10.1109/TMI.2016.2593957

164. Palmieri-Smith RM, Curran MT, Garcia SA, Krishnan C (2022) Factors That Predict Sagittal Plane Knee Biomechanical Symmetry After Anterior Cruciate Ligament Reconstruction: A Decision Tree Analysis. Sports Health 14:167–175. https://doi.org/10.1177/19417381211004932

165. Thomas P-A, Couderc A-L, Boulate D, Greillier L, Charvet A, Brioude G, Trousse D, D’Journo X-B, Barlesi F, Loundou A (2021) Early-stage non-small cell lung cancer beyond life expectancy: Still not too old for surgery? Lung Cancer 152:86–93. https://doi.org/10.1016/j.lungcan.2020.12.009

166. Drain A, Jun MS, Zhao LC (2021) Robotic Ureteral Reconstruction. Urologic Clinics of North America 48:91–101. https://doi.org/10.1016/j.ucl.2020.09.001

167. Wang R, Hacker MR, Richardson M (2021) Cost-effectiveness of Surgical Treatment Pathways for Prolapse. Female Pelvic Med Reconstr Surg 27:e408–e413. https://doi.org/10.1097/SPV.0000000000000948

168. Witjes JA, Bruins HM, Cathomas R, Compérat EM, Cowan NC, Gakis G, Hernández V, Linares Espinós E, Lorch A, Neuzillet Y, Rouanne M, Thalmann GN, Veskimäe E, Ribal MJ, van der Heijden AG (2021) European Association of Urology Guidelines on Muscle-invasive and Metastatic Bladder Cancer: Summary of the 2020 Guidelines. European Urology 79:82–104. https://doi.org/10.1016/j.eururo.2020.03.055

169. Spellman J, Coulter M, Kawatkar A, Calzada G (2020) Comparative cost of transoral robotic surgery and radiotherapy (IMRT) in early stage tonsil cancer. American Journal of Otolaryngology 41:102409. https://doi.org/10.1016/j.amjoto.2020.102409

170. Schmitt JJ, Baker MV, Occhino JA, McGree ME, Weaver AL, Bakkum-Gamez JN, Dowdy SC, Pasupathy KS, Gebhart JB (2020) Prospective Implementation and Evaluation of a Decision-Tree Algorithm for Route of Hysterectomy. Obstetrics & Gynecology 135:761–769. https://doi.org/10.1097/AOG.0000000000003725

171. Azari DP, Hu YH, Miller BL, Le BV, Radwin RG (2019) Using Surgeon Hand Motions to Predict Surgical Maneuvers. Hum Factors 61:1326–1339. https://doi.org/10.1177/0018720819838901

172. Martini A, Sfakianos JP, Paulucci DJ, Abaza R, Eun DD, Bhandari A, Hemal AK, Badani KK (2019) Predicting acute kidney injury after robot-assisted partial nephrectomy: Implications for patient selection and postoperative management. Urologic Oncology: Seminars and Original Investigations 37:445–451. https://doi.org/10.1016/j.urolonc.2019.04.018

173. Buse S, Hach CE, Klumpen P, Schmitz K, Mager R, Mottrie A, Haferkamp A (2018) Cost-effectiveness analysis of robot-assisted vs. open partial nephrectomy. Int J Med Robotics Comput Assist Surg 14:e1920. https://doi.org/10.1002/rcs.1920

174. Huben NB, Hussein AA, May PR, Whittum M, Krasowski C, Ahmed YE, Jing Z, Khan H, Kim HL, Schwaab T, Underwood W, Kauffman EC, Mohler JL, Guru KA (2018) Development of a Patient-Based Model for Estimating Operative Times for Robot-Assisted Radical Prostatectomy. Journal of Endourology 32:730–736. https://doi.org/10.1089/end.2018.0249

175. Hussein AA, May PR, Ahmed YE, Saar M, Wijburg CJ, Richstone L, Wagner A, Wilson T, Yuh B, Redorta JP, Dasgupta P, Kawa O, Khan MS, Menon M, Peabody JO, Hosseini A, Gaboardi F, Pini G, Schanne F, Mottrie A, Rha K, Hemal A, Stockle M, Kelly J, Tan WS, Maatman TJ, Poulakis V, Kaouk J, Canda AE, Balbay MD, Wiklund P, Guru KA (2017) Development of a patient and institutional-based model for estimation of operative times for robot-assisted radical cystectomy: results from the International Robotic Cystectomy Consortium. BJU Int 120:695–701. https://doi.org/10.1111/bju.13934

176. Schmitt JJ, Carranza Leon DA, Occhino JA, Weaver AL, Dowdy SC, Bakkum-Gamez JN, Pasupathy KS, Gebhart JB (2017) Determining Optimal Route of Hysterectomy for Benign Indications: Clinical Decision Tree Algorithm. Obstetrics & Gynecology 129:130–138. https://doi.org/10.1097/AOG.0000000000001756

177. Buse S, Hach CE, Klumpen P, Alexandrov A, Mager R, Mottrie A, Haferkamp A (2016) Cost-effectiveness of robot-assisted partial nephrectomy for the prevention of perioperative complications. World J Urol 34:1131–1137. https://doi.org/10.1007/s00345-015-1742-x

178. Rudmik L, An W, Livingstone D, Matthews W, Seikaly H, Scrimger R, Marshall D (2015) Making a case for high-volume robotic surgery centers: A cost-effectiveness analysis of transoral robotic surgery: Economics of Transoral Robotic Surgery. J Surg Oncol 112:155–163. https://doi.org/10.1002/jso.23974

179. Bailey JG, Hayden JA, Davis PJB, Liu RY, Haardt D, Ellsmere J (2014) Robotic versus laparoscopic Roux-en-Y gastric bypass (RYGB) in obese adults ages 18 to 65 years: a systematic review and economic analysis. Surg Endosc 28:414–426. https://doi.org/10.1007/s00464-013-3217-8

180. Martin AD, Nunez RN, Castle EP (2011) Robot-assisted Radical Cystectomy Versus Open Radical Cystectomy: A Complete Cost Analysis. Urology 77:621–625. https://doi.org/10.1016/j.urology.2010.07.502

181. Mao S, Yu X, Sun J, Yang Y, Shan Y, Sun J, Mugaanyi J, Fan R, Wu S, Lu C (2022) Development of nomogram models of inflammatory markers based on clinical database to predict prognosis for hepatocellular carcinoma after surgical resection. BMC Cancer 22:249. https://doi.org/10.1186/s12885-022-09345-2

182. Zhu H, Wang J, Gao T, Tian M, Xia L, Cai Q, Zhang C, Xu Y, Zheng X (2021) Contribution of revision amputation vs replantation for certain digits to functional outcomes after traumatic digit amputations: A comparative study based on multicenter prospective cohort. International Journal of Surgery 96:106164. https://doi.org/10.1016/j.ijsu.2021.106164

183. Naqvi SAA, Tennankore K, Vinson A, Roy PC, Abidi SSR (2021) Predicting Kidney Graft Survival Using Machine Learning Methods: Prediction Model Development and Feature Significance Analysis Study. J Med Internet Res 23:e26843. https://doi.org/10.2196/26843

184. Mirniaharikandehei S, Heidari M, Danala G, Lakshmivarahan S, Zheng B (2021) Applying a random projection algorithm to optimize machine learning model for predicting peritoneal metastasis in gastric cancer patients using CT images. Computer Methods and Programs in Biomedicine 200:105937. https://doi.org/10.1016/j.cmpb.2021.105937

185. Modaresnezhad M, Vahdati A, Nemati H, Ardestani A, Sadri F (2019) A rule-based semantic approach for data integration, standardization and dimensionality reduction utilizing the UMLS: Application to predicting bariatric surgery outcomes. Computers in Biology and Medicine 106:84–90. https://doi.org/10.1016/j.compbiomed.2019.01.019

186. Blum T, Feussner H, Navab N (2010) Modeling and segmentation of surgical workflow from laparoscopic video. Med Image Comput Comput Assist Interv 13:400–407. https://doi.org/10.1007/978-3-642-15711-0_50

187. Tronchot A, Berthelemy J, Thomazeau H, Huaulmé A, Walbron P, Sirveaux F, Jannin P (2021) Validation of virtual reality arthroscopy simulator relevance in characterising experienced surgeons. Orthopaedics & Traumatology: Surgery & Research 107:103079. https://doi.org/10.1016/j.otsr.2021.103079

188. Huaulmé A, Jannin P, Reche F, Faucheron J-L, Moreau-Gaudry A, Voros S (2020) Offline identification of surgical deviations in laparoscopic rectopexy. Artificial Intelligence in Medicine 104:101837. https://doi.org/10.1016/j.artmed.2020.101837

189. Peng W, Xing Y, Liu R, Li J, Zhang Z (2019) An automatic skill evaluation framework for robotic surgery training. Int J Med Robotics Comput Assist Surg 15:e1964. https://doi.org/10.1002/rcs.1964

190. Jiang J, Xing Y, Wang S, Liang K (2017) Evaluation of robotic surgery skills using dynamic time warping. Computer Methods and Programs in Biomedicine 152:71–83. https://doi.org/10.1016/j.cmpb.2017.09.007

191. Forestier G, Petitjean F, Riffaud L, Jannin P (2017) Automatic matching of surgeries to predict surgeons’ next actions. Artificial Intelligence in Medicine 81:3–11. https://doi.org/10.1016/j.artmed.2017.03.007

192. Forestier G, Lalys F, Riffaud L, Trelhu B, Jannin P (2012) Classification of surgical processes using dynamic time warping. Journal of Biomedical Informatics 45:255–264. https://doi.org/10.1016/j.jbi.2011.11.002

193. Zhang W, Sun M, Fan Y, Wang H, Feng M, Zhou S, Wang R (2021) Machine Learning in Preoperative Prediction of Postoperative Immediate Remission of Histology-Positive Cushing’s Disease. Front Endocrinol 12:635795. https://doi.org/10.3389/fendo.2021.635795

194. Baghdadi A, Hoshyarmanesh H, de Lotbiniere-Bassett MP, Choi SK, Lama S, Sutherland GR (2020) Data analytics interrogates robotic surgical performance using a microsurgery-specific haptic device. Expert Review of Medical Devices 17:721–730. https://doi.org/10.1080/17434440.2020.1782736

195. Miao R, Badger TC, Groesch K, Diaz-Sylvester PL, Wilson T, Ghareeb A, Martin JA, Cregger M, Welge M, Bushell C, Auvil L, Zhu R, Brard L, Braundmeier-Fleming A (2020) Assessment of peritoneal microbial features and tumor marker levels as potential diagnostic tools for ovarian cancer. PLoS ONE 15:e0227707. https://doi.org/10.1371/journal.pone.0227707

196. Nakawala H, Bianchi R, Pescatori LE, De Cobelli O, Ferrigno G, De Momi E (2019) “Deep-Onto” network for surgical workflow and context recognition. Int J CARS 14:685–696. https://doi.org/10.1007/s11548-018-1882-8

197. Egberts G, Schaaphok M, Vermolen F, Zuijlen P van (2022) A Bayesian finite-element trained machine learning approach for predicting post-burn contraction. Neural Comput & Applic. https://doi.org/10.1007/s00521-021-06772-3

198. Sargos P, Leduc N, Giraud N, Gandaglia G, Roumiguié M, Ploussard G, Rozet F, Soulié M, Mathieu R, Artus PM, Niazi T, Vinh-Hung V, Beauval J-B (2021) Deep Neural Networks Outperform the CAPRA Score in Predicting Biochemical Recurrence After Prostatectomy. Front Oncol 10:607923. https://doi.org/10.3389/fonc.2020.607923

199. Gleichgerrcht E, Keller SS, Drane DL, Munsell BC, Davis KA, Kaestner E, Weber B, Krantz S, Vandergrift WA, Edwards JC, McDonald CR, Kuzniecky R, Bonilha L (2020) Temporal Lobe Epilepsy Surgical Outcomes Can Be Inferred Based on Structural Connectome Hubs: A Machine Learning Study. Ann Neurol 88:970–983. https://doi.org/10.1002/ana.25888

200. Ştefănescu D, Streba C, Cârţână ET, Săftoiu A, Gruionu G, Gruionu LG (2016) Computer Aided Diagnosis for Confocal Laser Endomicroscopy in Advanced Colorectal Adenocarcinoma. PLoS ONE 11:e0154863. https://doi.org/10.1371/journal.pone.0154863

201. Yang G, Wang C, Yang J, Chen Y, Tang L, Shao P, Dillenseger J-L, Shu H, Luo L (2020) Weakly-supervised convolutional neural networks of renal tumor segmentation in abdominal CTA images. BMC Med Imaging 20:37. https://doi.org/10.1186/s12880-020-00435-w

202. DiPietro R, Ahmidi N, Malpani A, Waldram M, Lee GI, Lee MR, Vedula SS, Hager GD (2019) Segmenting and classifying activities in robot-assisted surgery with recurrent neural networks. Int J CARS 14:2005–2020. https://doi.org/10.1007/s11548-019-01953-x

203. Sachdeva N, Klopukh M, Clair RSt, Hahn WE (2021) Using conditional generative adversarial networks to reduce the effects of latency in robotic telesurgery. J Robotic Surg 15:635–641. https://doi.org/10.1007/s11701-020-01149-5

204. Wang Y, Zhou L, Wang M, Shao C, Shi L, Yang S, Zhang Z, Feng M, Shan F, Liu L (2020) Combination of generative adversarial network and convolutional neural network for automatic subcentimeter pulmonary adenocarcinoma classification. Quant Imaging Med Surg 10:1249–1264. https://doi.org/10.21037/qims-19-982

205. Chen L, Zhang F, Zhan W, Gan M, Sun L (2020) Optimization of virtual and real registration technology based on augmented reality in a surgical navigation system. BioMed Eng OnLine 19:1. https://doi.org/10.1186/s12938-019-0745-z

206. Mahmood F, Chen R, Durr NJ (2018) Unsupervised Reverse Domain Adaptation for Synthetic Medical Images via Adversarial Training. IEEE Trans Med Imaging 37:2572–2581. https://doi.org/10.1109/TMI.2018.2842767

207. van den Bosch T, Warps A-LK, de Nerée tot Babberich MPM, Stamm C, Geerts BF, Vermeulen L, Wouters MWJM, Dekker JWT, Tollenaar RAEM, Tanis PJ, Miedema DM, Dutch ColoRectal Audit (2021) Predictors of 30-Day Mortality Among Dutch Patients Undergoing Colorectal Cancer Surgery, 2011-2016. JAMA Netw Open 4:e217737. https://doi.org/10.1001/jamanetworkopen.2021.7737

208. López-Casado C, Bauzano E, Rivas-Blanco I, Pérez-del-Pulgar CJ, Muñoz VF (2019) A Gesture Recognition Algorithm for Hand-Assisted Laparoscopic Surgery. Sensors 19:5182. https://doi.org/10.3390/s19235182

209. Kim JW, Zhang P, Gehlbach P, Iordachita I, Kobilarov M (2021) Towards Autonomous Eye Surgery by Combining Deep Imitation Learning with Optimal Control. Proc Mach Learn Res 155:2347–2358

210. Zhou X-Y, Guo Y, Shen M, Yang G-Z (2020) Application of artificial intelligence in surgery. Front Med 14:417–430. https://doi.org/10.1007/s11684-020-0770-0

211. Tanwani AK, Sermanet P, Yan A, Anand R, Phielipp M, Goldberg K (2020) Motion2Vec: Semi-Supervised Representation Learning from Surgical Videos. In: 2020 IEEE International Conference on Robotics and Automation (ICRA). IEEE, Paris, France, pp 2174–2181

212. Montaña-Brown N, Ramalhinho J, Allam M, Davidson B, Hu Y, Clarkson MJ (2021) Vessel segmentation for automatic registration of untracked laparoscopic ultrasound to CT of the liver. Int J CARS 16:1151–1160. https://doi.org/10.1007/s11548-021-02400-6

213. Maier-Hein L, Wagner M, Ross T, Reinke A, Bodenstedt S, Full PM, Hempe H, Mindroc-Filimon D, Scholz P, Tran TN, Bruno P, Kisilenko A, Müller B, Davitashvili T, Capek M, Tizabi MD, Eisenmann M, Adler TJ, Gröhl J, Schellenberg M, Seidlitz S, Lai TYE, Pekdemir B, Roethlingshoefer V, Both F, Bittel S, Mengler M, Mündermann L, Apitz M, Kopp-Schneider A, Speidel S, Nickel F, Probst P, Kenngott HG, Müller-Stich BP (2021) Heidelberg colorectal data set for surgical data science in the sensor operating room. Sci Data 8:101. https://doi.org/10.1038/s41597-021-00882-2

214. Casella A, Moccia S, Paladini D, Frontoni E, De Momi E, Mattos LS (2021) A shape-constraint adversarial framework with instance-normalized spatio-temporal features for inter-fetal membrane segmentation. Medical Image Analysis 70:102008. https://doi.org/10.1016/j.media.2021.102008

215. Hattab G, Arnold M, Strenger L, Allan M, Arsentjeva D, Gold O, Simpfendörfer T, Maier-Hein L, Speidel S (2020) Kidney edge detection in laparoscopic image data for computer-assisted surgery: Kidney edge detection. Int J CARS 15:379–387. https://doi.org/10.1007/s11548-019-02102-0

216. Rosa B, Bordoux V, Nageotte F (2019) Combining Differential Kinematics and Optical Flow for Automatic Labeling of Continuum Robots in Minimally Invasive Surgery. Front Robot AI 6:86. https://doi.org/10.3389/frobt.2019.00086

217. Velazco Garcia JD, Navkar NV, Gui D, Morales CM, Christoforou EG, Ozcan A, Abinahed J, Al-Ansari A, Webb A, Seimenis I, Tsekos NV (2019) A Platform Integrating Acquisition, Reconstruction, Visualization, and Manipulator Control Modules for MRI-Guided Interventions. J Digit Imaging 32:420–432. https://doi.org/10.1007/s10278-018-0152-1

218. Shafiei SB, Durrani M, Jing Z, Mostowy M, Doherty P, Hussein AA, Elsayed AS, Iqbal U, Guru K (2021) Surgical Hand Gesture Recognition Utilizing Electroencephalogram as Input to the Machine Learning and Network Neuroscience Algorithms. Sensors 21:1733. https://doi.org/10.3390/s21051733

219. Shen J, Zemiti N, Taoum C, Aiche G, Dillenseger J-L, Rouanet P, Poignet P (2020) Transrectal ultrasound image-based real-time augmented reality guidance in robot-assisted laparoscopic rectal surgery: a proof-of-concept study. Int J CARS 15:531–543. https://doi.org/10.1007/s11548-019-02100-2

220. Feng Y, McGowan H, Semsar A, Zahiri HR, George IM, Turner T, Park A, Kleinsmith A, Mentis HM (2018) A virtual pointer to support the adoption of professional vision in laparoscopic training. Int J CARS 13:1463–1472. https://doi.org/10.1007/s11548-018-1792-9

221. Unger M, Black D, Fischer NM, Neumuth T, Glaser B (2019) Design and evaluation of an eye tracking support system for the scrub nurse. Int J Med Robotics Comput Assist Surg 15:e1954. https://doi.org/10.1002/rcs.1954

222. Cifuentes J, Pham MT, Boulanger P, Moreau R, Prieto F (2018) Gesture segmentation and classification using affine speed and energy. Proc Inst Mech Eng H 232:588–596. https://doi.org/10.1177/0954411918768350

223. Hou Y, Zhang W, Liu Q, Ge H, Meng J, Zhang Q, Wei X (2022) Adaptive kernel selection network with attention constraint for surgical instrument classification. Neural Comput & Applic 34:1577–1591. https://doi.org/10.1007/s00521-021-06368-x

224. Qiu H, Wang D, Miao H (2021) Analysis of the Effect of Robots in the Treatment of Pancreatic Cancer Based on Smart Medicine. Journal of Healthcare Engineering 2021:1–12. https://doi.org/10.1155/2021/9734882

225. Joelson A, Wildeman P, Sigmundsson FG, Rolfson O, Karlsson J (2021) Properties of the EQ-5D-5L when prospective longitudinal data from 28,902 total hip arthroplasty procedures are applied to different European EQ-5D-5L value sets. The Lancet Regional Health - Europe 8:100165. https://doi.org/10.1016/j.lanepe.2021.100165

226. Iseli T, Berghmans T, Glatzer M, Rittmeyer A, Massard G, Durieux V, Buchsbaum T, Putora PM (2020) Adverse events reporting in stage III NSCLC trials investigating surgery and radiotherapy. ERJ Open Res 6:00010–02020. https://doi.org/10.1183/23120541.00010-2020

227. Jung YJ, Seo HS, Lee HH, Kim JH, Song KY, Choi MH, Park CH (2018) Splenic Infarction as a Delayed Febrile Complication Following Radical Gastrectomy for Gastric Cancer Patients: Computed Tomography-Based Analysis. World J Surg 42:1826–1832. https://doi.org/10.1007/s00268-017-4401-0

228. Soguero-Ruiz C, Hindberg K, Mora-Jiménez I, Rojo-Álvarez JL, Skrøvseth SO, Godtliebsen F, Mortensen K, Revhaug A, Lindsetmo R-O, Augestad KM, Jenssen R (2016) Predicting colorectal surgical complications using heterogeneous clinical data and kernel methods. Journal of Biomedical Informatics 61:87–96. https://doi.org/10.1016/j.jbi.2016.03.008

229. Zhang G, Smith BP, Plate JF, Casanova R, Hsu F-C, Li J, Xia L, Li KC, Poehling GG, Zhou X (2016) A systematic approach to predicting the risk of unicompartmental knee arthroplasty revision. Osteoarthritis and Cartilage 24:991–999. https://doi.org/10.1016/j.joca.2016.01.004

230. Xue Q, Zhu Y, Wang Y, Yang J-J, Zhou C-M (2021) Using Nomograms to Predict the PPCs of Patients With Diffuse Peritonitis Undergoing Emergency Gastrointestinal Surgery. Front Med 8:705713. https://doi.org/10.3389/fmed.2021.705713

231. Shen X, Zhao H, Jin X, Chen J, Yu Z, Ramen K, Zheng X, Wu X, Shan Y, Bai J, Zhang Q, Zeng Q (2021) Development and validation of a machine learning-based nomogram for prediction of intrahepatic cholangiocarcinoma in patients with intrahepatic lithiasis. Hepatobiliary Surg Nutr 10:749–765. https://doi.org/10.21037/hbsn-20-332

232. Meng L, Zheng T, Wang Y, Li Z, Xiao Q, He J, Tan J (2021) Development of a prediction model based on LASSO regression to evaluate the risk of non-sentinel lymph node metastasis in Chinese breast cancer patients with 1–2 positive sentinel lymph nodes. Sci Rep 11:19972. https://doi.org/10.1038/s41598-021-99522-3

233. Kang J, Choi YJ, Kim I, Lee HS, Kim H, Baik SH, Kim NK, Lee KY (2021) LASSO-Based Machine Learning Algorithm for Prediction of Lymph Node Metastasis in T1 Colorectal Cancer. Cancer Res Treat 53:773–783. https://doi.org/10.4143/crt.2020.974

234. Shen J, Guo F, Sun Y, Zhao J, Hu J, Ke Z, Zhang Y, Jin X, Wu H (2021) Predictive nomogram for postoperative pancreatic fistula following pancreaticoduodenectomy: a retrospective study. BMC Cancer 21:550. https://doi.org/10.1186/s12885-021-08201-z

235. Aronsson L, Andersson R, Ansari D (2021) Artificial neural networks versus LASSO regression for the prediction of long-term survival after surgery for invasive IPMN of the pancreas. PLoS ONE 16:e0249206. https://doi.org/10.1371/journal.pone.0249206

236. Zhang Y, Zhu S, Yuan Z, Li Q, Ding R, Bao X, Zhen T, Fu Z, Fu H, Xing K, Yuan H, Chen T (2020) Risk factors and socio-economic burden in pancreatic ductal adenocarcinoma operation: a machine learning based analysis. BMC Cancer 20:1161. https://doi.org/10.1186/s12885-020-07626-2

237. Wu Y, Han C, Chong Y, Liu J, Gong L, Wang Z, Liang N (2020) Prognostic study for survival outcome following the treatment of second primary lung cancer in patients with previously resected non‐small cell lung cancer. Thorac Cancer 11:2840–2851. https://doi.org/10.1111/1759-7714.13610

238. Wang T, Yang X, Tang H, Kong J, Shen S, Qiu H, Wang W (2020) Integrated nomograms to predict overall survival and recurrence-free survival in patients with combined hepatocellular cholangiocarcinoma (cHCC) after liver resection. Aging 12:15334–15358. https://doi.org/10.18632/aging.103577

239. Zhao B, Gabriel RA, Vaida F, Eisenstein S, Schnickel GT, Sicklick JK, Clary BM (2020) Using machine learning to construct nomograms for patients with metastatic colon cancer. Colorectal Dis 22:914–922. https://doi.org/10.1111/codi.14991

240. Yang SU, Park EJ, Baik SH, Lee KY, Kang J (2019) Modified Colon Leakage Score to Predict Anastomotic Leakage in Patients Who Underwent Left-Sided Colorectal Surgery. JCM 8:1450. https://doi.org/10.3390/jcm8091450

241. Huang R, Xian S, Shi T, Yan P, Hu P, Yin H, Meng T, Huang Z (2019) Evaluating and Predicting the Probability of Death in Patients with Non-Metastatic Osteosarcoma: A Population-Based Study. Med Sci Monit 25:4675–4690. https://doi.org/10.12659/MSM.915418

242. Huang X, Liu J, Wu G, Chen S, Pc FJ, Xie W, Tang W (2019) Development and Validation of a Nomogram for Preoperative Prediction of Perineural Invasion in Colorectal Cancer. Med Sci Monit 25:1709–1717. https://doi.org/10.12659/MSM.914900

243. Zhao B, Waterman RS, Urman RD, Gabriel RA (2019) A Machine Learning Approach to Predicting Case Duration for Robot-Assisted Surgery. J Med Syst 43:32. https://doi.org/10.1007/s10916-018-1151-y

244. Klein G, Wang H, Elshabrawy A, Nashawi M, Gourley E, Liss M, Kaushik D, Wu S, Rodriguez R, Mansour AM (2021) Analyzing National Incidences and Predictors of Open Conversion During Minimally Invasive Partial Nephrectomy for cT1 Renal Masses. Journal of Endourology 35:30–38. https://doi.org/10.1089/end.2020.0161

245. Bhandari M, Nallabasannagari AR, Reddiboina M, Porter JR, Jeong W, Mottrie A, Dasgupta P, Challacombe B, Abaza R, Rha KH, Parekh DJ, Ahlawat R, Capitanio U, Yuvaraja TB, Rawal S, Moon DA, Buffi NM, Sivaraman A, Maes KK, Porpiglia F, Gautam G, Turkeri L, Meyyazhgan KR, Patil P, Menon M, Rogers C (2020) Predicting intra-operative and postoperative consequential events using machine-learning techniques in patients undergoing robot-assisted partial nephrectomy: a Vattikuti Collective Quality Initiative database study: Predicting events following RPN using ML. BJU Int 126:350–358. https://doi.org/10.1111/bju.15087

246. Cho H, Lee G, Lee HY, Park H (2020) Marginal radiomics features as imaging biomarkers for pathological invasion in lung adenocarcinoma. Eur Radiol 30:2984–2994. https://doi.org/10.1007/s00330-019-06581-2

247. Tosco L, Devos G, De Coster G, Roumeguère T, Everaerts W, Quackels T, Dekuyper P, Van Cleynenbreugel B, Van Damme N, Van Eycken E, Ameye F, Joniau S (2020) Development and external validation of a nomogram to predict lymph node invasion after robot assisted radical prostatectomy. Urologic Oncology: Seminars and Original Investigations 38:37.e11-37.e20. https://doi.org/10.1016/j.urolonc.2019.10.001

248. Murata T, Yanagisawa T, Kurihara T, Kaneko M, Ota S, Enomoto A, Tomita M, Sugimoto M, Sunamura M, Hayashida T, Kitagawa Y, Jinno H (2019) Salivary metabolomics with alternative decision tree-based machine learning methods for breast cancer discrimination. Breast Cancer Res Treat 177:591–601. https://doi.org/10.1007/s10549-019-05330-9

249. Kitahara H, McCrorey M, Patel B, Nisivaco S, Balkhy HH (2019) Predictors of blood transfusion use in robotic beating‐heart totally endoscopic coronary artery bypass with anastomotic connectors. J Card Surg 34:814–820. https://doi.org/10.1111/jocs.14150

250. Khene Z-E, Peyronnet B, Bernhard J-C, Kocher NJ, Vaessen C, Doumerc N, Pradere B, Seisen T, Beauval J-B, Verhoest G, Roumiguié M, De la Taille A, Bruyere F, Roupret M, Mejean A, Mathieu R, Shariat S, Raman JD, Bensalah K (2019) A preoperative nomogram to predict major complications after robot assisted partial nephrectomy (UroCCR-57 study). Urologic Oncology: Seminars and Original Investigations 37:577.e1-577.e7. https://doi.org/10.1016/j.urolonc.2019.05.007

251. Johnston SS, Morton JM, Kalsekar I, Ammann EM, Hsiao C-W, Reps J (2019) Using Machine Learning Applied to Real-World Healthcare Data for Predictive Analytics: An Applied Example in Bariatric Surgery. Value in Health 22:580–586. https://doi.org/10.1016/j.jval.2019.01.011

252. Baghdadi A, Hussein AA, Ahmed Y, Cavuoto LA, Guru KA (2019) A computer vision technique for automated assessment of surgical performance using surgeons’ console-feed videos. Int J CARS 14:697–707. https://doi.org/10.1007/s11548-018-1881-9

253. Sumathipala Y, Shafiq M, Bongen E, Brinton C, Paik D (2019) Machine learning to predict lung nodule biopsy method using CT image features: A pilot study. Computerized Medical Imaging and Graphics 71:1–8. https://doi.org/10.1016/j.compmedimag.2018.10.006

254. Wong NC, Lam C, Patterson L, Shayegan B (2019) Use of machine learning to predict early biochemical recurrence after robot-assisted prostatectomy. BJU Int 123:51–57. https://doi.org/10.1111/bju.14477

255. Fekri P, Dargahi J, Zadeh M (2021) Deep Learning-Based Haptic Guidance for Surgical Skills Transfer. Front Robot AI 7:586707. https://doi.org/10.3389/frobt.2020.586707

256. Psota E, Carlson J, Rodrigues Armijo P, Flores L, Siu K-C, Oleynikov D, Farritor S, Bills N (2021) End-Effector Contact and Force Detection for Miniature Autonomous Robots Performing Lunar and Expeditionary Surgery. Military Medicine 186:281–287. https://doi.org/10.1093/milmed/usaa443

257. Sun Y, Wang L, Jiang Z, Li B, Hu Y, Tian W (2020) State recognition of decompressive laminectomy with multiple information in robot-assisted surgery. Artificial Intelligence in Medicine 102:101763. https://doi.org/10.1016/j.artmed.2019.101763

258. Aviles AI, Alsaleh SM, Hahn JK, Casals A (2017) Towards Retrieving Force Feedback in Robotic-Assisted Surgery: A Supervised Neuro-Recurrent-Vision Approach. IEEE Trans Haptics 10:431–443. https://doi.org/10.1109/TOH.2016.2640289

259. Mourad M, Moubayed S, Dezube A, Mourad Y, Park K, Torreblanca-Zanca A, Torrecilla JS, Cancilla JC, Wang J (2020) Machine Learning and Feature Selection Applied to SEER Data to Reliably Assess Thyroid Cancer Prognosis. Sci Rep 10:5176. https://doi.org/10.1038/s41598-020-62023-w

260. Min JK, Kwak MS, Cha JM (2019) Overview of Deep Learning in Gastrointestinal Endoscopy. Gut and Liver 13:388–393. https://doi.org/10.5009/gnl18384

261. Mattfeldt T, Kestler HA, Hautmann R, Gottfried H-W (2001) Prediction of Postoperative Prostatic Cancer Stage on the Basis of Systematic Biopsies using Two Types of Artificial Neural Networks. Eur Urol 39:530–537. https://doi.org/10.1159/000052499

262. Köhler H, Kulcke A, Maktabi M, Moulla Y, Jansen-Winkeln B, Barberio M, Diana M, Gockel I, Neumuth T, Chalopin C (2020) Laparoscopic system for simultaneous high-resolution video and rapid hyperspectral imaging in the visible and near-infrared spectral range. J Biomed Opt 25:. https://doi.org/10.1117/1.JBO.25.8.086004

263. Zhang J, Gao X (2020) Object extraction via deep learning-based marker-free tracking framework of surgical instruments for laparoscope-holder robots. Int J CARS 15:1335–1345. https://doi.org/10.1007/s11548-020-02214-y

264. Tokuyasu T, Iwashita Y, Matsunobu Y, Kamiyama T, Ishikake M, Sakaguchi S, Ebe K, Tada K, Endo Y, Etoh T, Nakashima M, Inomata M (2021) Development of an artificial intelligence system using deep learning to indicate anatomical landmarks during laparoscopic cholecystectomy. Surg Endosc 35:1651–1658. https://doi.org/10.1007/s00464-020-07548-x

265. Loukas C, Varytimidis C, Rapantzikos K, Kanakis MA (2018) Keyframe extraction from laparoscopic videos based on visual saliency detection. Computer Methods and Programs in Biomedicine 165:13–23. https://doi.org/10.1016/j.cmpb.2018.07.004

266. Penza V, Du X, Stoyanov D, Forgione A, Mattos LS, De Momi E (2018) Long Term Safety Area Tracking (LT-SAT) with online failure detection and recovery for robotic minimally invasive surgery. Medical Image Analysis 45:13–23. https://doi.org/10.1016/j.media.2017.12.010

267. Feng W, Zhang Y, Liu W, Wang X, Lei T, Yuan Y, Chen Z, Song W (2022) A Prognostic Model Using Immune-Related Genes for Colorectal Cancer. Front Cell Dev Biol 10:813043. https://doi.org/10.3389/fcell.2022.813043

268. Lilo T, Morais CLM, Ashton KM, Davis C, Dawson TP, Martin FL, Alder J, Roberts G, Ray A, Gurusinghe N (2022) Raman hyperspectral imaging coupled to three-dimensional discriminant analysis: Classification of meningiomas brain tumour grades. Spectrochimica Acta Part A: Molecular and Biomolecular Spectroscopy 273:121018. https://doi.org/10.1016/j.saa.2022.121018

269. Vilmann AS, Svendsen MBS, Lachenmeier C, Søndergaard B, Vilmann P, Park YS, Svendsen LB, Konge L (2022) Colonoscope retraction technique and predicting adenoma detection rate: a multicenter study. Gastrointestinal Endoscopy S0016510721019404. https://doi.org/10.1016/j.gie.2021.12.026

270. Song H, Ruan C, Xu Y, Xu T, Fan R, Jiang T, Cao M, Song J (2021) Survival stratification for colorectal cancer via multi-omics integration using an autoencoder-based model. Exp Biol Med (Maywood) 153537022110650. https://doi.org/10.1177/15353702211065010

271. Li Z, Li Z, Chen Q, Ramos A, Zhang J, Boudreaux JP, Thiagarajan R, Bren-Mattison Y, Dunham ME, McWhorter AJ, Li X, Feng J-M, Li Y, Yao S, Xu J (2021) Detection of pancreatic cancer by convolutional-neural-network-assisted spontaneous Raman spectroscopy with critical feature visualization. Neural Networks 144:455–464. https://doi.org/10.1016/j.neunet.2021.09.006

272. Wang Z, Zou W, Wang F, Zhang G, Chen K, Hu M, Liu R (2021) Identification of the immune cell infiltration landscape in pancreatic cancer to assist immunotherapy. Future Oncology 17:4131–4143. https://doi.org/10.2217/fon-2021-0495

273. Yocum D, Reinbolt J, Weinhandl JT, Standifird TW, Fitzhugh E, Cates H, Zhang S (2021) Principal Component Analysis of Knee Joint Differences Between Bilateral and Unilateral Total Knee Replacement Patients During Level Walking. Journal of Biomechanical Engineering 143:111003. https://doi.org/10.1115/1.4051524

274. Ebina K, Abe T, Higuchi M, Furumido J, Iwahara N, Kon M, Hotta K, Komizunai S, Kurashima Y, Kikuchi H, Matsumoto R, Osawa T, Murai S, Tsujita T, Sase K, Chen X, Konno A, Shinohara N (2021) Motion analysis for better understanding of psychomotor skills in laparoscopy: objective assessment-based simulation training using animal organs. Surg Endosc 35:4399–4416. https://doi.org/10.1007/s00464-020-07940-7

275. Aruse O, Immerman I, Badir O, Haj ME, Volk I, Luria S (2021) Scaphoid fracture displacement is not correlated with the fracture angle. J Hand Surg Eur Vol 46:607–615. https://doi.org/10.1177/17531934211004434

276. Lin X-H, Yang U-C, Luo J-C, Chang T-E, Lin H-H, Huang C-W, Chiou J-J, Fang W-L, Huang K-H, Huang Y-H, Hou M-C, Lee F-Y (2021) Differences in intestinal microbiota profiling after upper and lower gastrointestinal surgery. Journal of the Chinese Medical Association 84:354–360. https://doi.org/10.1097/JCMA.0000000000000510

277. Lee MJ, Jones GL, Lobo AJ, Brown SR, the pCD collaborators, Bethune R, Blackmore A, Fearnhead N, Guy R, Hancock L, Hill J, Moran G, Pinkney T, Verjee A, Williams A, Wilson T (2021) Survey to define informational needs of patients undergoing surgery for Crohn’s anal fistula. Colorectal Dis 23:132–144. https://doi.org/10.1111/codi.15423

278. Berl Q, Resseguier N, Katsogiannou M, Mauviel F, Carcopino X, Boubli L, Blanc J (2021) Objective assessment of obstetrics residents’ surgical skills in caesarean: Development and evaluation of a specific rating scale. Journal of Gynecology Obstetrics and Human Reproduction 50:101812. https://doi.org/10.1016/j.jogoh.2020.101812

279. Zhou Z, Wang Y, Niu Y, He Z, Huang M, Zhou Y, Lv W, Hu J (2020) How we assess the perioperative anxiety of surgical patients with pulmonary nodules: the revision of state-trait anxiety inventory. J Cardiothorac Surg 15:324. https://doi.org/10.1186/s13019-020-01338-1

280. Alizadeh Savareh B, Asadzadeh Aghdaie H, Behmanesh A, Bashiri A, Sadeghi A, Zali M, Shams R (2020) A machine learning approach identified a diagnostic model for pancreatic cancer through using circulating microRNA signatures. Pancreatology 20:1195–1204. https://doi.org/10.1016/j.pan.2020.07.399

281. Noori SMR, Farnia P, Bayat M, Bahrami N, Shakourirad A, Ahmadian A (2020) Automatic detection of symmetry plane for computer-aided surgical simulation in craniomaxillofacial surgery. Phys Eng Sci Med 43:1087–1099. https://doi.org/10.1007/s13246-020-00909-9

282. Schlosser KA, Maloney SR, Prasad T, Colavita PD, Augenstein VA, Heniford BT (2020) Three-dimensional hernia analysis: the impact of size on surgical outcomes. Surg Endosc 34:1795–1801. https://doi.org/10.1007/s00464-019-06931-7

283. Drijkoningen T, Mohamadi A, Luria S, Buijze GA (2019) Scaphoid Fracture Patterns—Part One: Three-Dimensional Computed Tomography Analysis. Jnl Wrist Surg 08:441–445. https://doi.org/10.1055/s-0039-1693050

284. Zúñiga WC, Jones V, Anderson SM, Echevarria A, Miller NL, Stashko C, Schmolze D, Cha PD, Kothari R, Fong Y, Storrie-Lombardi MC (2019) Raman Spectroscopy for Rapid Evaluation of Surgical Margins during Breast Cancer Lumpectomy. Sci Rep 9:14639. https://doi.org/10.1038/s41598-019-51112-0

285. Jeng M-J, Sharma M, Sharma L, Chao T-Y, Huang S-F, Chang L-B, Wu S-L, Chow L (2019) Raman Spectroscopy Analysis for Optical Diagnosis of Oral Cancer Detection. JCM 8:1313. https://doi.org/10.3390/jcm8091313

286. Lin X-H, Jiang J-K, Luo J-C, Lin C-C, Ting P-H, Yang U-C, Lan Y-T, Huang Y-H, Hou M-C, Lee F-Y (2019) The long term microbiota and metabolic status in patients with colorectal cancer after curative colon surgery. PLoS ONE 14:e0218436. https://doi.org/10.1371/journal.pone.0218436

287. Gündoğdu Y, Alptekin H, Karabağlı P, Şahin M, Kilic HŞ (2019) Discrimination of cancerous and healthy colon tissues: A new laser‐based method. Lasers Surg Med 51:363–369. https://doi.org/10.1002/lsm.23033

288. Brotons A, Guilabert M, Lacueva F, Mira J, Lumbreras B, Picó M, Vitaller J, García-Sepulcre M, Belda G, Sola-Vera J (2019) The Colonoscopy Satisfaction and Safety Questionnaire (CSSQP) for Colorectal Cancer Screening: A Development and Validation Study. IJERPH 16:392. https://doi.org/10.3390/ijerph16030392

289. Catanuto G, Taher W, Rocco N, Catalano F, Allegra D, Milotta FLM, Stanco F, Gallo G, Nava MB (2019) Breast Shape Analysis With Curvature Estimates and Principal Component Analysis for Cosmetic and Reconstructive Breast Surgery. Aesthetic Surgery Journal 39:164–173. https://doi.org/10.1093/asj/sjy070

290. Abelson JS, Sosa JA, Symer MM, Mao J, Michelassi F, Bell R, Sedrakyan A, Yeo HL (2018) Association of Expectations of Training With Attrition in General Surgery Residents. JAMA Surg 153:712. https://doi.org/10.1001/jamasurg.2018.0611

291. Oropesa I, Escamirosa FP, Sánchez-Margallo JA, Enciso S, Rodríguez-Vila B, Martínez AM, Sánchez-Margallo FM, Gómez EJ, Sánchez-González P (2018) Interpretation of motion analysis of laparoscopic instruments based on principal component analysis in box trainer settings. Surg Endosc 32:3096–3107. https://doi.org/10.1007/s00464-018-6022-6

292. Phelps DL, Balog J, Gildea LF, Bodai Z, Savage A, El-Bahrawy MA, Speller AV, Rosini F, Kudo H, McKenzie JS, Brown R, Takáts Z, Ghaem-Maghami S (2018) The surgical intelligent knife distinguishes normal, borderline and malignant gynaecological tissues using rapid evaporative ionisation mass spectrometry (REIMS). Br J Cancer 118:1349–1358. https://doi.org/10.1038/s41416-018-0048-3

293. Dubert T, Girault C, Kilink A, Rozenblat M, Lebellec Y, Vataire A-L, Vilasco M, Katz G (2017) Development of an instrument evaluating the impact of surgeon-patient relationship in patients on sick leave. Journal of Market Access & Health Policy 5:1345586. https://doi.org/10.1080/20016689.2017.1345586

294. Pennell C, Polet C, Arthur LG, Grewal H, Aronoff S (2020) Risk assessment for intra-abdominal injury following blunt trauma in children: Derivation and validation of a machine learning model. J Trauma Acute Care Surg 89:153–159. https://doi.org/10.1097/TA.0000000000002717

295. Wu G, Woodruff HC, Sanduleanu S, Refaee T, Jochems A, Leijenaar R, Gietema H, Shen J, Wang R, Xiong J, Bian J, Wu J, Lambin P (2020) Preoperative CT-based radiomics combined with intraoperative frozen section is predictive of invasive adenocarcinoma in pulmonary nodules: a multicenter study. Eur Radiol 30:2680–2691. https://doi.org/10.1007/s00330-019-06597-8

296. Yang S, Yoon H, Yazdi SJM, Lee J (2020) A novel automated lumen segmentation and classification algorithm for detection of irregular protrusion after stents deployment. Int J Med Robot 16:. https://doi.org/10.1002/rcs.2033

297. Hung AJ, Chen J, Che Z, Nilanon T, Jarc A, Titus M, Oh PJ, Gill IS, Liu Y (2018) Utilizing Machine Learning and Automated Performance Metrics to Evaluate Robot-Assisted Radical Prostatectomy Performance and Predict Outcomes. Journal of Endourology 32:438–444. https://doi.org/10.1089/end.2018.0035

298. Su H, Hu Y, Karimi HR, Knoll A, Ferrigno G, De Momi E (2020) Improved recurrent neural network-based manipulator control with remote center of motion constraints: Experimental results. Neural Networks 131:291–299. https://doi.org/10.1016/j.neunet.2020.07.033

299. Chen T, Liu S, Li Y, Feng X, Xiong W, Zhao X, Yang Y, Zhang C, Hu Y, Chen H, Lin T, Zhao M, Liu H, Yu J, Xu Y, Zhang Y, Li G (2019) Developed and validated a prognostic nomogram for recurrence-free survival after complete surgical resection of local primary gastrointestinal stromal tumors based on deep learning. EBioMedicine 39:272–279. https://doi.org/10.1016/j.ebiom.2018.12.028

300. Sefati S, Gao C, Iordachita I, Taylor RH, Armand M (2021) Data-Driven Shape Sensing of a Surgical Continuum Manipulator Using an Uncalibrated Fiber Bragg Grating Sensor. IEEE Sensors J 21:3066–3076. https://doi.org/10.1109/JSEN.2020.3028208

301. Vidal F, Guerby P, Simon C, Lesourd F, Cartron G, Parinaud J, Tanguy le Gac Y, Dupuis N (2021) Spontaneous pregnancy rate following surgery for deep infiltrating endometriosis in infertile women: The impact of the learning curve. Journal of Gynecology Obstetrics and Human Reproduction 50:101942. https://doi.org/10.1016/j.jogoh.2020.101942

302. Zeuschner P, Meyer I, Siemer S, Stoeckle M, Wagenpfeil G, Wagenpfeil S, Saar M, Janssen M (2021) Three Different Learning Curves Have an Independent Impact on Perioperative Outcomes After Robotic Partial Nephrectomy: A Comparative Analysis. Ann Surg Oncol 28:1254–1261. https://doi.org/10.1245/s10434-020-08856-1

303. Mortani Barbosa EJ, Sachs N (2021) CT Fluoroscopy Guided Thoracic Biopsies (CTTB) Are Highly Accurate and Safe: Outcomes and Predictive Modeling of Complications Utilizing Machine Learning. Academic Radiology 28:608–618. https://doi.org/10.1016/j.acra.2020.03.036

304. Luzzago S, Rosiello G, Pecoraro A, Deuker M, Stolzenbach F, Mistretta FA, Tian Z, Musi G, Montanari E, Shariat SF, Saad F, Briganti A, de Cobelli O, Karakiewicz PI (2020) Contemporary Rates and Predictors of Open Conversion During Minimally Invasive Radical Prostatectomy for Nonmetastatic Prostate Cancer. Journal of Endourology 34:600–607. https://doi.org/10.1089/end.2020.0074

305. Bajpai RR, Razdan S, Sanchez-Gonzalez MA, Razdan S (2020) Retrospective Cohort Analysis from a High-Volume Center of Prognostic Factors Affecting Biochemical Relapse in Patients with Encapsulated, Margin-Negative, Isolated Seminal Vesicle Invasion After Robot-Assisted Laparoscopic Prostatectomy: A Novel Study. Journal of Endourology 34:441–449. https://doi.org/10.1089/end.2019.0714

306. Semrau JS, Scott SH, Hamilton AG, Petsikas D, Payne DM, Bisleri G, Saha T, Boyd JG (2020) Quantified pre-operative neurological dysfunction predicts outcome after coronary artery bypass surgery. Aging Clin Exp Res 32:289–297. https://doi.org/10.1007/s40520-019-01184-9

307. Poelaert F, Joniau S, Roumeguère T, Ameye F, De Coster G, Dekuyper P, Quackels T, Van Cleynenbreugel B, Van Damme N, Van Eycken E, Mottrie A, Lumen N (2019) Current Management of pT3b Prostate Cancer After Robot-assisted Laparoscopic Prostatectomy. European Urology Oncology 2:110–117. https://doi.org/10.1016/j.euo.2018.05.005

308. Oquendo YA, Riddle EW, Hiller D, Blinman TA, Kuchenbecker KJ (2018) Automatically rating trainee skill at a pediatric laparoscopic suturing task. Surg Endosc 32:1840–1857. https://doi.org/10.1007/s00464-017-5873-6

309. Hussein AA, Ahmed YE, May P, Ali T, Ahmad B, Raheem S, Stone K, Hasasnah A, Rana O, Cole A, Wang D, Loud P, Guru KA (2018) Natural History and Predictors of Parastomal Hernia after Robot-Assisted Radical Cystectomy and Ileal Conduit Urinary Diversion. Journal of Urology 199:766–773. https://doi.org/10.1016/j.juro.2017.08.112

310. Hyldgård VB, Laursen KR, Poulsen J, Søgaard R (2017) Robot-assisted surgery in a broader healthcare perspective: a difference-in-difference-based cost analysis of a national prostatectomy cohort. BMJ Open 7:e015580. https://doi.org/10.1136/bmjopen-2016-015580

311. Sexton K, Johnson A, Gotsch A, Hussein AA, Cavuoto L, Guru KA (2018) Anticipation, teamwork and cognitive load: chasing efficiency during robot-assisted surgery. BMJ Qual Saf 27:148–154. https://doi.org/10.1136/bmjqs-2017-006701

312. Brown JD, O’Brien CE, Leung SC, Dumon KR, Lee DI, Kuchenbecker KJ (2017) Using Contact Forces and Robot Arm Accelerations to Automatically Rate Surgeon Skill at Peg Transfer. IEEE Trans Biomed Eng 64:2263–2275. https://doi.org/10.1109/TBME.2016.2634861

313. Addae JK, Gani F, Fang SY, Wick EC, Althumairi AA, Efron JE, Canner JK, Euhus DM, Schneider EB (2017) A comparison of trends in operative approach and postoperative outcomes for colorectal cancer surgery. Journal of Surgical Research 208:111–120. https://doi.org/10.1016/j.jss.2016.09.019

314. Xu W, Chen J, Lau HYK, Ren H (2017) Data-driven methods towards learning the highly nonlinear inverse kinematics of tendon-driven surgical manipulators: XWJ_IJMRAS_IK_KNNR_GMR_ELM. Int J Med Robotics Comput Assist Surg 13:e1774. https://doi.org/10.1002/rcs.1774

315. Lei Y, Bao S, Wang J (2016) The combined effects of action observation and passive proprioceptive training on adaptive motor learning. Neuroscience 331:91–98. https://doi.org/10.1016/j.neuroscience.2016.06.011

316. Potretzke AM, Potretzke TA, Knight BA, Vetter J, Park AM, Anderson G, Bhayani SB, Figenshau RS (2016) Tumor diameter accurately predicts perioperative outcomes in T1 renal cancer treated with robot-assisted partial nephrectomy. World J Urol 34:1643–1650. https://doi.org/10.1007/s00345-016-1809-3

317. Pavan N, Autorino R, Lee H, Porpiglia F, Sun Y, Greco F, Jeff Chueh S, Han DH, Cindolo L, Ferro M, Chen X, Branco A, Fornara P, Liao C-H, Miyajima A, Kyriazis I, Puglisi M, Fiori C, Yang B, Fei G, Altieri V, Jeong BC, Berardinelli F, Schips L, De Cobelli O, Chen Z, Haber G-P, He Y, Oya M, Liatsikos E, Brandao L, Challacombe B, Kaouk J, Darweesh I (2016) Impact of novel techniques on minimally invasive adrenal surgery: trends and outcomes from a contemporary international large series in urology. World J Urol 34:1473–1479. https://doi.org/10.1007/s00345-016-1791-9

318. Yoon AP, Wang Y, Wang L, Chung KC, and the WRIST Group (2021) What Are the Tradeoffs in Outcomes after Casting Versus Surgery for Closed Extraarticular Distal Radius Fractures in Older Patients? A Statistical Learning Model. Clin Orthop Relat Res 479:2691–2700. https://doi.org/10.1097/CORR.0000000000001865

319. Wu L, He X, Liu M, Xie H, An P, Zhang J, Zhang H, Ai Y, Tong Q, Guo M, Huang M, Ge C, Yang Z, Yuan J, Liu J, Zhou W, Jiang X, Huang X, Mu G, Wan X, Li Y, Wang H, Wang Y, Zhang H, Chen D, Gong D, Wang J, Huang L, Li J, Yao L, Zhu Y, Yu H (2021) Evaluation of the effects of an artificial intelligence system on endoscopy quality and preliminary testing of its performance in detecting early gastric cancer: a randomized controlled trial. Endoscopy 53:1199–1207. https://doi.org/10.1055/a-1350-5583

320. Sakamoto T, Goto T, Fujiogi M, Kawarai Lefor A (2021) Machine learning in gastrointestinal surgery. Surg Today. https://doi.org/10.1007/s00595-021-02380-9

321. Byerly S, Maurer LR, Mantero A, Naar L, An G, Kaafarani HMA (2021) Machine Learning and Artificial Intelligence for Surgical Decision Making. Surgical Infections 22:626–634. https://doi.org/10.1089/sur.2021.007

322. Ostberg NP, Zafar MA, Elefteriades JA (2021) Machine learning: principles and applications for thoracic surgery. European Journal of Cardio-Thoracic Surgery 60:213–221. https://doi.org/10.1093/ejcts/ezab095

323. Datta S, Li Y, Ruppert MM, Ren Y, Shickel B, Ozrazgat-Baslanti T, Rashidi P, Bihorac A (2021) Reinforcement learning in surgery. Surgery 170:329–332. https://doi.org/10.1016/j.surg.2020.11.040

324. Solanki SL, Pandrowala S, Nayak A, Bhandare M, Ambulkar RP, Shrikhande SV (2021) Artificial intelligence in perioperative management of major gastrointestinal surgeries. WJG 27:2758–2770. https://doi.org/10.3748/wjg.v27.i21.2758

325. Liang X, Yang X, Yin S, Malay S, Chung KC, Ma J, Wang K (2021) Artificial Intelligence in Plastic Surgery: Applications and Challenges. Aesth Plast Surg 45:784–790. https://doi.org/10.1007/s00266-019-01592-2

326. Ma R, Vanstrum EB, Lee R, Chen J, Hung AJ (2020) Machine learning in the optimization of robotics in the operative field. Current Opinion in Urology 30:808–816. https://doi.org/10.1097/MOU.0000000000000816

327. Loftus TJ, Tighe PJ, Filiberto AC, Balch J, Upchurch GR, Rashidi P, Bihorac A (2020) Opportunities for machine learning to improve surgical ward safety. The American Journal of Surgery 220:905–913. https://doi.org/10.1016/j.amjsurg.2020.02.037

328. Loftus TJ, Filiberto AC, Li Y, Balch J, Cook AC, Tighe PJ, Efron PA, Upchurch GR, Rashidi P, Li X, Bihorac A (2020) Decision analysis and reinforcement learning in surgical decision-making. Surgery 168:253–266. https://doi.org/10.1016/j.surg.2020.04.049

329. Du Z, Wang W, Yan Z, Dong W, Wang W (2017) Variable Admittance Control Based on Fuzzy Reinforcement Learning for Minimally Invasive Surgery Manipulator. Sensors 17:844. https://doi.org/10.3390/s17040844

330. Trovato G, Shikanai M, Ukawa G, Kinoshita J, Murai N, Lee JW, Ishii H, Takanishi A, Tanoue K, Ieiri S, Konishi K, Hashizume M (2010) Development of a colon endoscope robot that adjusts its locomotion through the use of reinforcement learning. Int J CARS 5:317–325. https://doi.org/10.1007/s11548-010-0481-0

331. Gessert N, Bengs M, Schlüter M, Schlaefer A (2020) Deep learning with 4D spatio-temporal data representations for OCT-based force estimation. Medical Image Analysis 64:101730. https://doi.org/10.1016/j.media.2020.101730

332. Zhao W, Yang J, Sun Y, Li C, Wu W, Jin L, Yang Z, Ni B, Gao P, Wang P, Hua Y, Li M (2018) 3D Deep Learning from CT Scans Predicts Tumor Invasiveness of Subcentimeter Pulmonary Adenocarcinomas. Cancer Res 78:6881–6889. https://doi.org/10.1158/0008-5472.CAN-18-0696

333. Hsiao V, Elfenbein DM, Pitt SC, Long KL, Sippel RS, Schneider DF (2022) Evaluating Discrimination of ACS-NSQIP Surgical Risk Calculator in Thyroidectomy Patients. Journal of Surgical Research 271:137–144. https://doi.org/10.1016/j.jss.2021.10.016

334. Choi J, Marafino BJ, Vendrow EB, Tennakoon L, Baiocchi M, Spain DA, Forrester JD (2021) Rib Fracture Frailty Index: A Risk-Stratification Tool for Geriatric Patients with Multiple Rib Fractures. Journal of Trauma and Acute Care Surgery Publish Ahead of Print: https://doi.org/10.1097/TA.0000000000003390

335. Ai Y, Zhang J, Jin J, Zhang J, Zhu H, Jin X (2021) Preoperative Prediction of Metastasis for Ovarian Cancer Based on Computed Tomography Radiomics Features and Clinical Factors. Front Oncol 11:610742. https://doi.org/10.3389/fonc.2021.610742

336. Fulton L, Kruse CS (2019) Hospital-Based Back Surgery: Geospatial-Temporal, Explanatory, and Predictive Models. J Med Internet Res 21:e14609. https://doi.org/10.2196/14609

337. Pan B, Zhang G, Xia JJ, Yuan P, Ip HHS, He Q, Lee PKM, Chow B, Zhou X (2016) Prediction of soft tissue deformations after CMF surgery with incremental kernel ridge regression. Computers in Biology and Medicine 75:1–9. https://doi.org/10.1016/j.compbiomed.2016.04.020

338. Sánchez-Peralta LF, Picón A, Sánchez-Margallo FM, Pagador JB (2020) Unravelling the effect of data augmentation transformations in polyp segmentation. Int J CARS 15:1975–1988. https://doi.org/10.1007/s11548-020-02262-4

339. Hwang S-J, Park S-J, Kim G-M, Baek J-H (2021) Unsupervised Monocular Depth Estimation for Colonoscope System Using Feedback Network. Sensors 21:2691. https://doi.org/10.3390/s21082691

340. Kelly JD, Nash M, Heller N, Lendvay TS, Kowalewski TM (2020) Temporal variability of surgical technical skill perception in real robotic surgery. Int J CARS 15:2101–2107. https://doi.org/10.1007/s11548-020-02253-5

341. Aminsharifi A, Irani D, Tayebi S, Jafari Kafash T, Shabanian T, Parsaei H (2020) Predicting the Postoperative Outcome of Percutaneous Nephrolithotomy with Machine Learning System: Software Validation and Comparative Analysis with Guy’s Stone Score and the CROES Nomogram. Journal of Endourology 34:692–699. https://doi.org/10.1089/end.2019.0475

342. Chen T, Zhang C, Liu Y, Zhao Y, Lin D, Hu Y, Yu J, Li G (2019) A gastric cancer LncRNAs model for MSI and survival prediction based on support vector machine. BMC Genomics 20:846. https://doi.org/10.1186/s12864-019-6135-x

343. Ershad M, Rege R, Majewicz Fey A (2019) Automatic and near real-time stylistic behavior assessment in robotic surgery. Int J CARS 14:635–643. https://doi.org/10.1007/s11548-019-01920-6

344. Quero G, Lapergola A, Barberio M, Seeliger B, Saccomandi P, Guerriero L, Mutter D, Saadi A, Worreth M, Marescaux J, Agnus V, Diana M (2019) Discrimination between arterial and venous bowel ischemia by computer-assisted analysis of the fluorescent signal. Surg Endosc 33:1988–1997. https://doi.org/10.1007/s00464-018-6512-6

345. Rafii-Tari H, Payne CJ, Bicknell C, Kwok K-W, Cheshire NJW, Riga C, Yang G-Z (2017) Objective Assessment of Endovascular Navigation Skills with Force Sensing. Ann Biomed Eng 45:1315–1327. https://doi.org/10.1007/s10439-017-1791-y

346. Fujiyoshi K, Väyrynen JP, Borowsky J, Papke DJ, Arima K, Haruki K, Kishikawa J, Akimoto N, Ugai T, Lau MC, Gu S, Shi S, Zhao M, Da Silva AFL, Twombly TS, Nan H, Meyerhardt JA, Song M, Zhang X, Wu K, Chan AT, Fuchs CS, Lennerz JK, Giannakis M, Nowak JA, Ogino S (2020) Tumour budding, poorly differentiated clusters, and T-cell response in colorectal cancer. EBioMedicine 57:102860. https://doi.org/10.1016/j.ebiom.2020.102860

347. Eshelman MA, Jeganathan NA, Schieffer KM, Kline BP, Mendenhall M, Deiling S, Harris L, Koltun WA, Yochum GS (2019) Elevated Colonic Mucin Expression Correlates with Extended Time to Surgery for Ulcerative Colitis Patients. JGLD 28:405–413. https://doi.org/10.15403/jgld-250

348. Tepas JJ, Kerwin AJ, de Villa J, Ra JH, Nussbaum MS (2016) Restating Surgical Risk: From Patient to Population. Journal of the American College of Surgeons 222:505–512. https://doi.org/10.1016/j.jamcollsurg.2015.12.023

349. Xu K, Chen Z, Jia F (2019) Unsupervised binocular depth prediction network for laparoscopic surgery. Computer Assisted Surgery 24:30–35. https://doi.org/10.1080/24699322.2018.1557889

350. Clay R, Rajagopalan S, Karwoski R, Maldonado F, Peikert T, Bartholmai B (2018) Computer Aided Nodule Analysis and Risk Yield (CANARY) characterization of adenocarcinoma: radiologic biopsy, risk stratification and future directions. Transl Lung Cancer Res 7:313–326. https://doi.org/10.21037/tlcr.2018.05.11
